# Molecular basis of an agarose metabolic pathway acquired by a human intestinal symbiont

**DOI:** 10.1038/s41467-018-03366-x

**Published:** 2018-03-13

**Authors:** Benjamin Pluvinage, Julie M. Grondin, Carolyn Amundsen, Leeann Klassen, Paul E. Moote, Yao Xiao, Dallas Thomas, Nicholas A. Pudlo, Anuoluwapo Anele, Eric C. Martens, G. Douglas Inglis, Richard E. R. Uwiera, Alisdair B. Boraston, D. Wade Abbott

**Affiliations:** 1 Department of Biochemistry and Microbiology, University of Victoria, PO Box 3055 STN CSC, Victoria, BC V8W 3P6 Canada; 2 Agriculture and Agri-Food Canada, Lethbridge Research and Development Centre, Lethbridge, AB T1J 4B1 Canada; 3 Department of Agricultural, Food and Nutritional Science, University of Alberta, Edmonton, AB T6G 2P5 Canada; 4 Department of Microbiology and Immunology, University of Michigan Medical School, Ann Arbor, MI 48109 USA

## Abstract

In red algae, the most abundant principal cell wall polysaccharides are mixed galactan agars, of which agarose is a common component. While bioconversion of agarose is predominantly catalyzed by bacteria that live in the oceans, agarases have been discovered in microorganisms that inhabit diverse terrestrial ecosystems, including human intestines. Here we comprehensively define the structure–function relationship of the agarolytic pathway from the human intestinal bacterium *Bacteroides uniformis* (*Bu*) NP1. Using recombinant agarases from *Bu* NP1 to completely depolymerize agarose, we demonstrate that a non-agarolytic *Bu* strain can grow on GAL released from agarose. This relationship underscores that rare nutrient utilization by intestinal bacteria is facilitated by the acquisition of highly specific enzymes that unlock inaccessible carbohydrate resources contained within unusual polysaccharides. Intriguingly, the agarolytic pathway is differentially distributed throughout geographically distinct human microbiomes, reflecting a complex historical context for agarose consumption by human beings.

## Abstract (web summary)

Polysaccharides are the primary structural cell wall and energy storage molecules of seaweed. Here, the authors show how the geographically restricted dietary polysaccharide agarose is selectively utilized by the human intestinal bacterium *Bacteroides uniformis*, providing insight into how carbohydrate metabolism evolves within the human microbiome.

## Introduction

Consuming seaweed has been a practice of the coastal peoples of Far East Asia since antiquity. In 600 bc, the Chinese author Sze Teu was quoted as saying “Some algae are a delicacy fit for the most honored guests, even for the King himself” (anonymous). Over a century later in medieval Europe (ad 563), Saint Columba, a monk who founded the Island of Iona monastery, proclaimed that “…plucking dulse (*Palmaria palmata*) from the rocks…” was a cherished pastime^1^. The commercial viability of algal polysaccharides has since grown into a $1.1B global industry with applications that extend beyond the food industry. For example, algal cell walls are widely considered a viable option for the production of renewable bioproducts^2^.

Polysaccharides are the primary structural cell wall and energy storage molecules of seaweed. In red algae, the most abundant glycans are mixed galactan agars, of which agarose, porphyran, and carrageenan are common components. Agarose has a linear backbone with repeating disaccharide subunits of 3-*O*-β-d-galactose (GAL) and 4-*O-*α-3,6-anhydro-l-galactose (AHG). Porphyran is structurally related to agarose, with the exception that the AHG subunit is replaced with 4-*O-*α-l-galactose-6-sulfate (L6S). Similarly, carrageenan also comprises repeating α-1,3-linked disaccharides of GAL β-1,4 linked to another GAL residue. However, the carrageenan family is structurally diverse, with the alternating GAL residue being either GAL or 4-*O-*α-3,6-anhydro-d-galactose, and displaying varying degrees of sulfation at C2, C4, and C6. The anhydrous residues of agars are not found in land plants and require tailored glycoside hydrolases (GHs) for enzymatic turnover. Furthermore, the presence of sulfations and hydroxymethylations alter the rheological properties of agars, and dictate the formation of higher order structures that modulate the physical properties of the algal cell wall. Despite the significance of these polysaccharides for marine ecosystems, our understanding of the molecular basis of their utilization lags far behind what is known about land plant polysaccharides, such as cellulose, xylan, and pectin.

The bioconversion of algal polysaccharides in the ocean is rapid and highly efficient, and represents fundamental biochemical processes for the cycling of nutrients in the biosphere^3^. Correspondingly, the largest reservoir of agarolytic genes is found within marine ecosystems, and marine bacteria responsible for the bioconversion of algae tend to have genomes enriched in carbohydrate active enzyme (CAZyme) genes belonging to hallmark agarase GH families (e.g., GH50, GH86, GH117^3,4^). Intriguingly, agarases have been discovered in the genomes of microorganisms that inhabit fresh water^5,6^, soil^7–9^, and human intestines^10^. This suggests that some agarolytic microbes may have evolved specialized pathways to support a nomadic lifestyle or enable them to consume displaced nutrients that permeate their local microenvironments.

A general pathway for agarose saccharification (agarolysis) by marine microorganisms has been established^11–14^. Initial depolymerization occurs in the extracellular environment as agar is a large heterogeneous network that is refractory to transport. The upstream reactions are catalyzed by *endo*-acting agarases, such as GH16s^15–17^ and GH86s^18–20^, that hydrolyze internal β-1,4 linkages and release a range of neoagarooligosaccharides (NAOS). Terminal β-1,4 linkages at the non-reducing end of NAOS are cleaved by *exo-*acting GH50s^21,22^ or GH86Es^13^, which exclusively release the disaccharide neoagarobiose (N2; AHG-α-1,3-GAL). This product is hydrolyzed by *exo-*acting α-1,3-agarases from family GH117 into AHG and GAL monosaccharides prior to or after transport, depending upon the agarolytic system. More recently, an alternative pathway from the marine bacterium *Vibrio sp*. EJY3 has been described that harnesses a GH2 β-galactosidase to remove a single GAL at the non-reducing end of agarooligosaccharides (AOS)^23,24^. AOS are generated by *endo-*acting α-agarases (GH96), which are extremely rare, or *exo-*acting GH117s^24^. Very little is currently known about the mechanistic role of sulfatases and demethylases in agar saccharification.

In phylum Bacteroidetes, CAZymes that operate systematically to dismantle a defined carbohydrate substrate are organized into genetic clusters called polysaccharide utilization loci (PULs)^25,26^. In intestinal *Bacteroides* spp., these systems are generally tailored for dietary complex carbohydrates that are indigestible to human enzymes (i.e., dietary fiber). In addition to associated enzyme activities, PULs often contain transport machinery (SusC/D-like systems) and regulatory proteins (e.g., hybrid-two component systems (HTCSs))^27,28^. The detection of coregulated genes within PULs has been transformational for enzyme discovery and has accelerated the characterization of CAZymes with rare or unknown functions and of unconventional saccharification pathways^29,30^. For example, the presence of a functional porphyran PUL in the human intestinal bacterium *Bacteroides plebeius* (*Bp*) DSM 17135 was recently reported^31^. Acquisition of this pathway was postulated to have resulted from horizontal transfer of an integrative conjugative element that originated from a marine bacterium. The presence of the porphyran PUL was observed to be abundant in Japanese individuals that consume *Porphyra* spp. in their diets^32^. Recently, other algae-metabolizing intestinal bacteria have been discovered, including the carrageenolytic *Bacteroides thetaiotaomicron* VPI-3731, agarolytic *Bu* NP1, and alginolytic *Bacteroides ovatus*^33^, suggesting there are more universal diet–host–microbe relationships driving the coevolution of algal galactan metabolism within the intestinal microbiome^31,32^. The nutrient-niche hypothesis of Freter et al.^34^ predicts that ecological niches within the intestine are defined by nutrient availability, and selectively occupying these niches may provide a fitness advantage for intestinal bacteria^35^. Therefore, defining the interactions between the intestinal microbiome and rare or geographically restricted polysaccharides, such as agarose, provides unique insights into how nutrients are selectively metabolized within a competitive ecosystem. Overall, these findings contribute to a growing paradigm shift in our understanding of how the distal gut microbiome adapts to the chemical diversity of indigestible dietary carbohydrates.

## Results

### Delineation of agarolytic genes

**Fig. 1:**
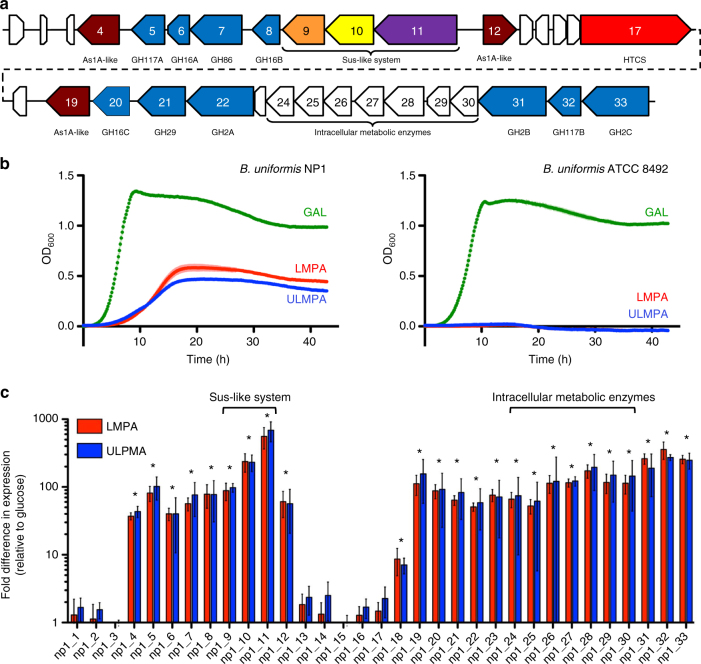
Functional agarolysis is *Bu* NP1. **a** Schematic representation of the Ag-PUL drawn to relative size and with orientations illustrated with arrows. Genes are labeled from 1 to 33 (*np1*_1–33) and according to their predicted functions. Brown = sulfatases, blue = GHs, orange = SusE-like, yellow = SusD-like, purple = SusC-like, red = response regulator, and white = hypothetical proteins. Genes are labeled according to their CAZy classification. **b** Growth curves of agarolytic *Bu* NP1 (left panel) and non-agarolytic *Bu* ATCC 8492 (right panel) on GAL, LMPA, and ULMPA. The experiment was replicated three times, with four to eight observations per replicate; error (s.d.) is represented by trace thickness. **c** RT-PCR of the genes in the Ag-PUL. Relative expression (normalized to glucose) compared to HK genes is shown; statistical significance is represented as *p < 0.05. Vertical lines associated with histogram error bars (s.e.m.) represent three biological replicates, with six independent measurements per replicate

The genome of *Bu* NP1 was analyzed for the presence of hallmark agarase genes using the dbCAN server for the annotation of translated CAZyme sequences^36^, and for full open reading frame (ORF) sequences using BLASTp^37^ and Artemis^38^. A genetic segment of approximately 49.5 kbp encoding 33 predicted genes (*np1*_1 to *np1*_33) was identified that appears to have inserted into a GH5-encoding gene (BACUNI_ 03803) of an ancestral non-agarolytic *Bu* strain (Fig. 1a; Supplementary Table 1). The segment contains three GH2s (GH2A–C) and GH16s (GH16A–C), two GH117s (GH117A and B), and one GH29 and GH86. The presence of GH16s, GH117s, and a GH86 is consistent with an anticipated activity on algal galactans. In addition, the PUL contains three putative sulfatases and a cluster of *sus*-like genes, entitled *susC*-, *susD*-, and *susE*-like. The Sus system was first identified in the starch utilization system of *B. thetaiotaomicron*^39^ and refers to the archetypical outer membrane TonB-dependent system responsible for the depolymerization and import of dedicated complex carbohydrate substrates^25^. Additionally, the segment contains a putative HTCS transcriptional regulator, which is a family of sensory proteins known to induce the expression of PULs^27,28^. Therefore, the genomic sequence spanning *np1_*1 to *np1_*33 was identified as the putative agarolytic PUL in *Bu* NP1 (Ag-PUL).

### Identification of a cytoplasmic pathway for AHG metabolism

Complete saccharification of agarose results in the generation of GAL and AHG monosaccharides. GAL is a ubiquitous nutrient for environmental bacteria and enters glycolysis following epimerization by the Leloir pathway^40^. In contrast, AHG is a rare dietary carbohydrate that contains an intra-ring anhydrous bridge not found in terrestrial plants, and requires an alternative set of reactions for conversion into conventional metabolic intermediates. For example, in the marine bacterium *Vibrio* sp. EJY3 a two-step pathway converts AHG into 3,6-anhydrogalactonate by AHG dehydrogenase (AHGD), which is then converted to 2-keto-3-deoxygalactonate by AHG isomerase (AHGI)^2,41^. Comparisons of the translated gene sequences within the Ag-PUL reveal that the NP1*_*27 and NP1*_*28 proteins show high levels of similarity (>64%) to AHGI and AHGD (Supplementary Table 2), respectively. Putative functions for several other genes not commonly associated with traditional PUL systems were also detected in the Ag-PUL. These include a contiguous segment of genes with homology to intracellular metabolic enzymes, encoding a putative ketodeoxygluconate kinase (KdgK, *np1_*24), galactose mutarotase (GalM, *np1_*26), and a RhaT-like sugar importer (*np1_*25) (Fig. 1a, Supplementary Table 1). It is plausible that these gene products may be responsible for the metabolism of AHG, which is a known metabolic attribute of marine agarolytic bacteria^41^. In this regard, the import of monosaccharide products and their subsequent bioconversion into chemical intermediates appear to be catalyzed by core metabolic enzymes that are co-regulated with agarose depolymerization, suggesting that energy extraction from agarose requires enzyme activities not encoded within the core genome of that *Bu*, that *Bu* NP1 can utilize both GAL and AHG during growth, and that total agarose catabolism appears to have been acquired by an en bloc horizontal transfer event.

### Activation of the Ag-PUL

To ascertain if *Bu* NP1 could grow on multiple agarose sources with different structures and solubility profiles, cells were cultured on low melting point agarose (LMPA) and ultra-low melting point agarose (ULMPA). The difference between these two polysaccharides is that, as a result of higher levels of methylation, ULMPA has a lower gelation temperature (8–17 °C) than LMPA (~25 °C). Continuously monitored growth kinetics demonstrated that *Bu* NP1 grows on both forms of agarose (LMPA > ULMPA), but at a slower rate and to a lower density compared to GAL (Fig. 1b). The increased growth of *Bu* NP1 on LMPA compared to ULMPA suggests that unmethylated agarose residues may be preferentially metabolized; indeed, a putative demethylase was not identified as part of the Ag-PUL. In comparison, *Bu* ATCC 8492, which lacks a conserved Ag-PUL sequence, did not grow on either agarose substrate (Fig. 1b).

To determine if the predicted agarolytic pathway was activated during the metabolism of agarose, reverse transcription-PCR (RT-PCR) was performed for all 33 predicted genes in the pathway (Supplementary Table 3). Amplicons were normalized using validated housekeeping genes (Supplementary Fig. 1; Supplementary Table 4). There was a significant increase (*p* < 0.05) in the expression of 25 of 33 genes when cells were grown on LMPA and ULMPA compared to glucose (Fig. 1c); however, there was no difference between growth in the LMPA and ULMPA cultures. This observation suggests that the entire pathway is upregulated by a similar mechanism, most likely the single predicted HTCS in the PUL (*np1*_17). Induced genes were clustered in two major blocks (*np1*_4–12 and *np1*_18–33) with the majority of genes having a predicted function consistent with the PUL-mediated metabolism of agarose. These include all 3 sulfatases, 10 GHs, and the Sus-like system. Additionally, the predicted intracellular pathway (*np1*_24–30) and two hypothetical protein genes (*np1*_18 and *np1*_23) were induced. The HTCS regulator (*np1*_17) was not upregulated, which is consistent with other reported PULs^42^. Increases in expression levels for *np1*_*1–33* ranged from ~10-fold (*np1*_18) to 560-fold (*np1*_11).

### Functional characterization of the agarases from the Ag-PUL

**Fig. 2:**
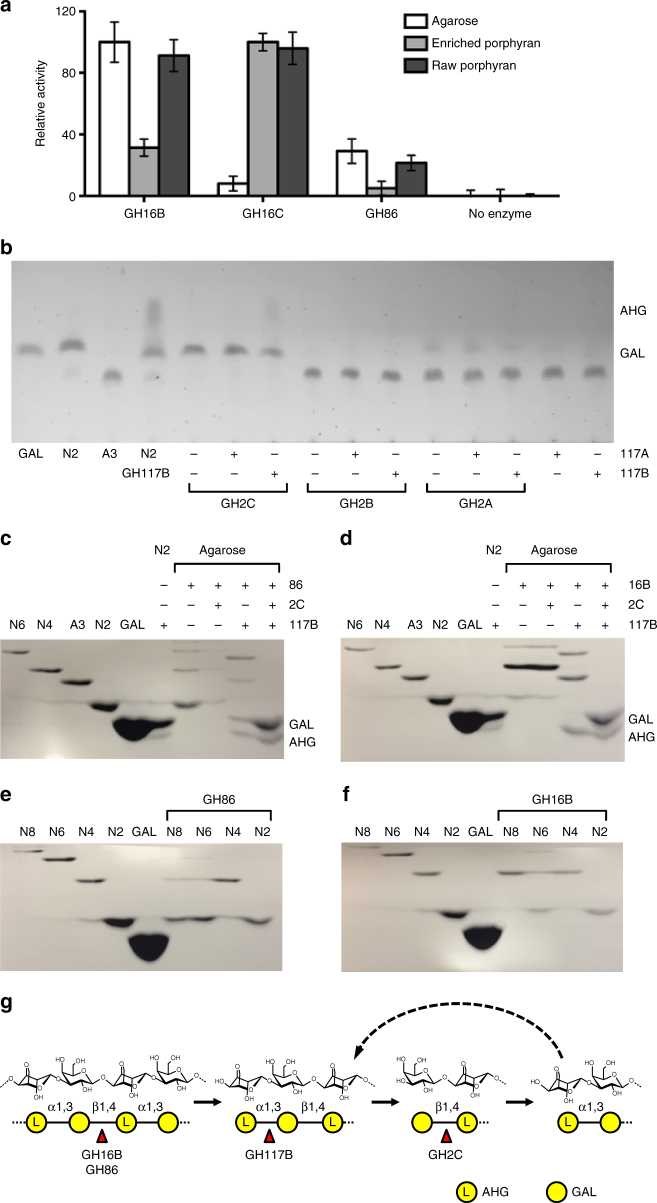
Agarolytic reaction pathway of *Bu* NP1 agarases. **a** Quantification of GH16B, GH16C, and GH86 activity using agarose, raw porphyran, and enriched porphyran. Error (s.d.) is represented by error bars over three replicates. **b** TLC analysis of the *exo-*activity of GH117s and GH2s. FACE analysis of agarose digestion with **c** GH86-containing and **d** GH16B-containing reaction mixtures. FACE analysis of NAOS digestion with **e** GH86-containing and **f** GH16B-containing reaction mixtures. **g** Schematic of the agarolytic reaction pathway. The β-1,4 linkages of agarose and large NAOS are hydrolyzed by GH86 and GH16B to release smaller NAOS fragments. Next, the newly exposed α-1,3-linked AHG is cleaved from the non-reducing end by GH117B to generate AOS products, which are substrates for GH2C. GH2C hydrolyzes the β-1,4-linked galactose at the non-reducing end to regenerate NAOS with a degree of polymerization that has collectively been reduced by a single disaccharide. Depolymerization is cyclical and is repeated by alternating reaction steps with GH117B and GH2C

The putative GHs in the Ag-PUL belonging to GH families previously associated with agarolytic activity (i.e., GH2, GH16, GH86, and GH117 enzymes) were targeted for molecular cloning and biochemical characterization (Supplementary Table 5). Of these, GH16A, GH16B, GH16C, and GH86 fall into families containing members with *endo-*hydrolytic activities on agarose, porphyran, or κ-carrageenan, and therefore, these four enzymes were screened by reducing sugar assay against these substrates. Initial screens demonstrated that GH16A had no detectable activity on the tested substrates, while GH16C had activity only on porphyran, and GH16B and GH86 showed activity on both agarose and porphyran. While agarose is largely a homogeneous polysaccharide, native porphyran is a heteropolymer that also contains segments of agarose^43^. Therefore, the activity of GH16B, GH16C, and GH86 were quantified on agarose, porphyan, and enriched porphyran (predigested with agarases) (Fig. 2a). There was a noticeable drop in activity of GH16B and GH86 on enriched porphyran compared to untreated porphyran, whereas GH16C retained full activity on both substrates, a pattern of activity typical of porphyranases^31^. These results indicate that GH16B and GH86 are conventional agarases and GH16C is a porphyranase (Supplementary Table 6). The observation that each enzyme is induced by agarose (Fig. 1c) suggests that the efficient digestion of natural algal substrates (e.g., dulse) requires the combinatorial activities of agarases and porphyranses.

GH117 is a relatively small CAZyme family primarily found in marine bacteria^4^. Presently, characterized members of this family include α-1,3-L-neoagarooligosaccharide hydrolases (EC 3.2.1.) that remove AHG from the non-reducing end of agars or N2^44–47^. The lack of a hallmark GH50 or GH86E, enzymes that are known to release N2 from agarose in an *exo*-hydrolytic fashion, in the Ag-PUL suggests that an alternate route would be required for complete saccharification of agarose by *Bu* NP1. Recently, a novel agarolytic GH2 β-galactosidase was discovered in the marine bacterium *Vibrio* sp. EJY3^24^. This enzyme possesses sequence homology to the GH2s from the Ag-PUL of *Bu* NP1 (GH2C = 42.7%, GH2A = 29.8%, GH2B = 19.9%; Supplementary Fig. 2). Thus, we hypothesized that GH2A, GH2B, and GH2C comprise putative agarose-specific *exo-*acting β-d-galactosidases, while GH117A and GH117B are α-1,3-L-neoagarooligosaccharide hydrolases. These enzymes were screened on agarotriose (A3; GAL-β-1,4-AHG-α-1,3-GAL) (Fig. 2b). GH2C was able to hydrolyze A3, suggesting that GH2C is an *exo*-β-1,4-galactosidase. Notably, the other enzymes did not display activity on this substrate. Co-treatment of A3 with GH2C and GH117B, but not GH117A, resulted in a new set of products (Fig. 2b) identical to GAL and AHG released from N2 by GH117B (Fig. 2b). Thus, GH2C and GH117B are agarose-specific *exo*-β-d-galactosidase and an *exo*-α-1,3-L-neoagarooligosaccharide hydrolase, respectively. The activities of GH2B, GH2A, and GH117A remain unknown as we found no evidence of their activity on agarose.

Having identified the key agarolytic enzymes in the Ag-PUL, the products generated by *endo-*acting β-agarases GH16B and GH86 were examined in further detail. Both enzymes generated a ladder of products from insoluble agarose, with GH86 predominantly producing a product that resembled N2 and GH16B producing an N4 product (Fig. 2c, d); these activities correspond to those reported for related GH86s^20,48^ and GH16s^49,50^. Digestion of NAOS substrates to completion with GH86 and GH16B verified this conclusion, and revealed subtle differences between the subsite architecture of both enzymes (Fig. 2e, f). GH86 was able to digest neoagarooctaose (N8) and neoagarohexaose (N6) to N2, but exhibited no activity on neoagarotetraose (N4) (Fig. 2e). GH16B predominantly generated N4 from N8, N2 and N4 from N6, but exhibited no activity on N4 (Fig. 2f). Although GH86 and GH16B have similar overall specificities, the production of distinct product profiles from agarose and NAOS highlight that they possess unique active site subsite compositions responsible for the generation of products with varying degrees of polymerization. The presence of functionally related enzymes from different GH families present in the agarolytic pathway suggests the enzymes may be trafficked to alternative compartments within the bacterial cell, where the generation of differential product profiles may be mandatory or advantageous in vivo. In this manner, GH86 is predicted to be secreted into the periplasm, whereas GH16B has an outer membrane targeting peptide (Supplementary Table 1).

Each of the two *endo-*acting β-agarases was screened for activity on agarose in combination with the GH2C *exo*-β-d-agarase, and/or with the GH117B *exo*-α-l-agarase (Fig. 2c, d). Consistent with the exposure of new AHG residues at the non-reducing end following treatment of agarose with GH86 or GH16B, co-treatment with GH117B but not GH2C resulted in the generation of a new set of products, likely AOS produced by the removal of AHG residues at the non-reducing termini of NAOS. Combinatorial digestions of agarose with GH2C, GH117B, and either GH86 or GH16B resulted in the generation of GAL and AHG monosaccharides, and the disappearance of AOS and NAOS (Fig. 2c, d, Supplementary Fig. 3). This suggests that GH117B and GH2C are co-dependent *exo-*acting enzymes that act cyclically on newly exposed monosaccharides at the non-reducing ends of NAOS and AOS^23^, respectively.

Together, the functional studies conducted have defined an agarolytic pathway comprising GH86, GH16B, GH117B, and GH2C as the key components (Fig. 2g). Agarose digestion is initiated by GH86 or GH16B, which generates NAOS. These products are then cyclically degraded into the constituent monosaccharides AHG and GAL by the coordinated removal of these monosaccharides from the non-reducing end of NAOS and AOS by GH117B and GH2C, respectively. In order to further elucidate the molecular basis of agarolysis by the Ag-PUL of *Bu* NP1, we determined the three-dimensional structures of these four agarases.

### The molecular basis of *Bu* NP1 *endo-*acting agarases

**Fig. 3:**
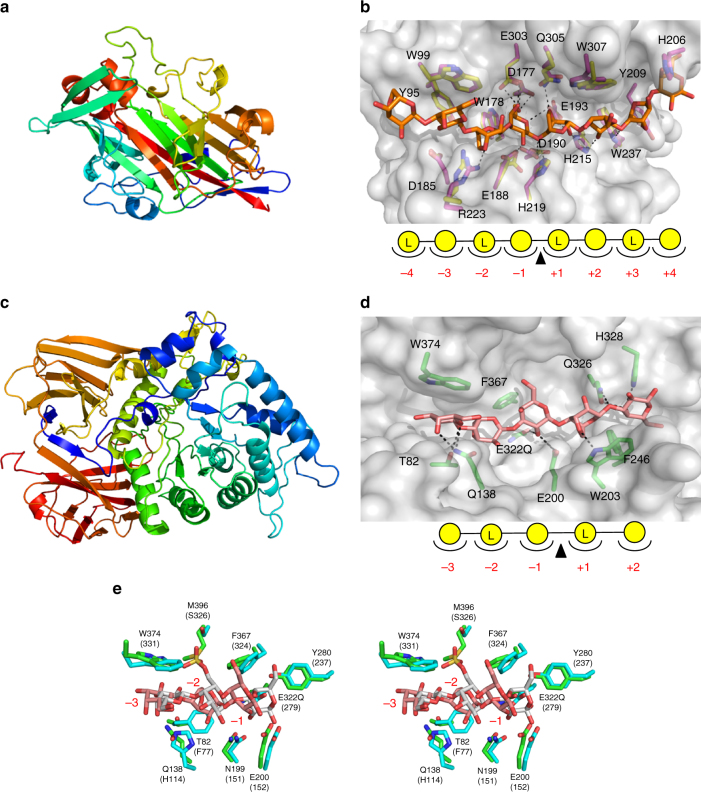
Molecular basis of *endo-*acting agarases from *Bu* NP1. **a** Structure of GH16B shown in cartoon format color ramped from N terminus (blue) to C terminus (red). **b** Surface representation (gray) of the GH16B catalytic groove. Key GH16B residues, shown as numbered purple sticks and labeled, are overlapped with structurally conserved residues from *Z. galactanivorans* AgaB (yellow) in complex with N8 (orange) (pdb entry: 4ATF^17^). The subsites are indicated below and hydrogen bonds are represented by dashed lines. **c** Structure of GH86 shown in cartoon format color ramped from N terminus (blue) to C terminus (red). **d** Surface representation (gray) of the GH86 active site in complex with N8 (modeled residues for A5 shown in pink). Key residues are numbered and shown in green. The subsites are indicated below and hydrogen bonds are represented by dashed lines. **e** Wall-eyed stereo view of the −1, −2, and −3 subsites using the structural alignment of GH86 (green) in complex with N8 (pink) with *Bp*GH86A (cyan) in complex with a porphyran/agarose hexasaccharide (gray) (pdb entry 4AW7^31^). Key GH86 residues are numbered, with corresponding *Bp*GH86 residues in brackets

The structure of GH16B (2.5 Å) was determined using the agarase *Bp*GH16B (pdb entry: 2AWD^31^) as a molecular replacement model (Fig. 3a). GH16B from *Bu* displays the β-jelly roll fold typical of the GH16 family. Comparison of this structure to that of AgaB from *Zobellia galactanivorans*, which was determined in complex with N8, gave an amino acid identity of 48.7% and a root mean square deviation of 0.966 Å over 266 matched Cα atoms. Furthermore, the architecture of the AgaB active site was highly conserved with the predicted active site of GH16B, suggesting the presence of eight active site subsites in GH16B, indicated as plus (+) and minus (−) subsites (Fig. 3b). This is consistent with the thin layer chromatography (TLC) and fluorophore-assisted carbohydrate electrophoresis (FACE) results, which showed the enzyme preferentially produces N4 from agarose, to completely convert N8 to N4, and to have weak activity on N6 (Fig. 2d, f).

The structure of GH86 (2.3 Å) was determined using the porphyranase *Bp*GH86A (pdb entry: 4AW7^31^) as a molecular replacement model. The GH86 possesses a core (α/β)_8_ barrel with two Ig-like domains (Fig. 3c). An inactive E322Q nucleophile mutant of GH86 was generated, and the structure in complex with N8 was determined to a resolution of 1.4 Å (Fig. 3d, Supplementary Fig. 4a). This generated clear electron density for an oligosaccharide that spanned the catalytic machinery in the active site (Supplementary Fig. 4b). Although the ligand used was N8, only an agaropentaose (A5) molecule occupying subsites −3 to +2 could be modeled, suggesting the presence of five subsites in the active site of GH86 (Fig. 3d, Supplementary Fig. 4a). Notably, the sugars occupying the +1 and +2 subsites correspond to N2, which is thus consistent with the ability of this enzyme to release predominantly N2 from agarose and NAOS (Fig. 2c, e).

The catalytic residues identified in *Bp*GH86A were conserved in GH86 despite differences in the specificities between these two enzymes (Fig. 3e, Supplementary Fig. 4c). A comparison of the carbohydrate-bound structures of GH86 and *Bp*GH86A, where the bound carbohydrates overlap at the −1 to −3 subsites, provided valuable insights into the molecular basis of differential agarose and porphyran specificities within these two GH86 family members (Fig. 3e). GH86 and *Bp*GH86A employ very similar modes of carbohydrate recognition in their −1 and −3 subsites, which are occupied by GAL residues. *Bp*GH86A is a product complex while the GH86 structure is a Michaelis-like complex, explaining the different positions of the GAL residues in the −1 subsite. However, despite accounting for the E322Q mutation in GH86, the complement of amino acid sidechains directly interacting with the GAL residue in the −1 subsite was identical. Likewise, the −3 subsite in both enzymes was largely defined by a conserved tryptophan residue that formed a classic CH–π interaction with the GAL residue in this subsite. An additional amino acid sidechain in each enzyme (Q138 in GH86 and H114 in *Bp*GH86A) was structurally conserved and likely is responsible for similar hydrogen bonding interactions (Fig. 3e). In contrast, the −2 subsite was quite divergent and likely responsible in large part for the selectivity of the enzymes for agarose versus porphyran. In *Bp*GH86A, this subsite was occupied by a L6S residue, which introduces a kink into the sugar (Fig. 3e). The space created by the kink was occupied by the phenyl ring of F77 while the 6-sulfate group projects into a pocket, at the base of which is a serine residue (S326). In contrast, the −2 subsite of GH86 was occupied by a α-linked AHG residue that, by virtue of its two-membered ring structure, was in a confirmation similar to that of a β-linked d-sugar. As such, the trajectory of A5 in the active site of GH86 was different than that of porphyran in *Bp*GH86A; the sugar was accommodated by a threonine residue (T82) in the place of the phenylalanine residue (F77). Conversely, the pocket occupied by the sulfate group in *Bp*GH86A was filled by a methionine sidechain in GH86 (M396), which effectively replaces the serine residue in *Bp*GH86A. Together, subsite −2 differentially selected for the conformation and chemical properties imparted by the polysaccharide substrate, namely L6S in porphyran or AGH in agarose.

### The molecular basis of *Bu* NP1 *exo-*acting agarases

**Fig. 4:**
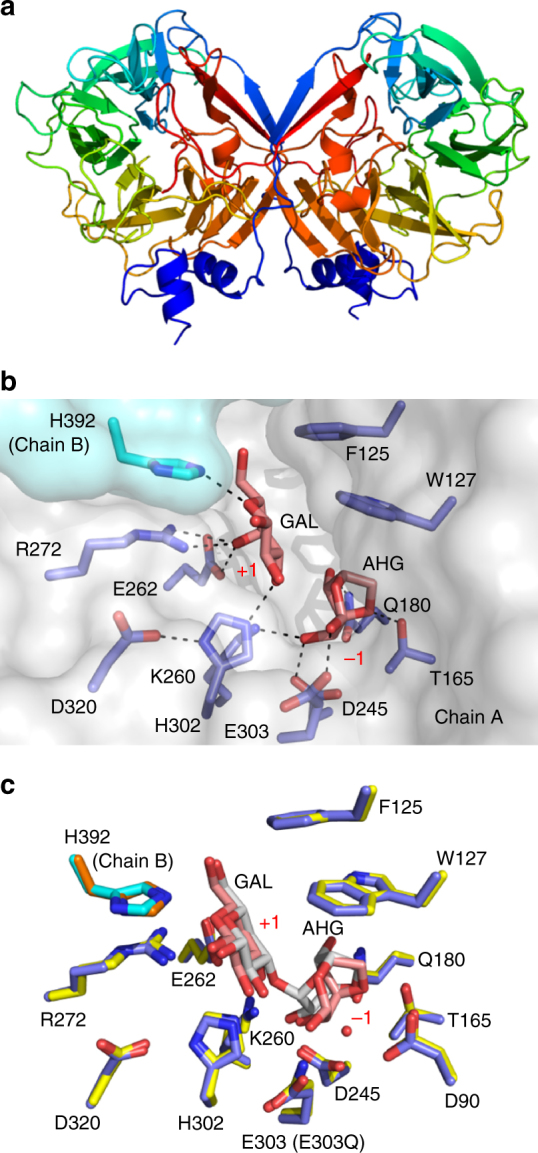
Molecular basis of the *exo-*acting α-1,3-l-neoagarooligosaccharide hydrolase GH117B. **a** Structure of the GH117B dimer shown in cartoon format with each monomer color ramped from N terminus (blue) to C terminus (red). **b** Surface representation of chain A (gray) and chain B (cyan) of the GH117B catalytic pocket. Key residues are labeled (blue) and N2 digestion products GAL and AHG are shown in stick format (pink). **c** Structural alignment of GH117B (blue) in complex with GAL and AHG (pink) with *Bp*GH117 (yellow) in complex with N2 (gray) (pdb entry 4AK5^47^). Numbering for conserved GH117B and *Bp*GH117 residues is shown, and when different, indicated in brackets for the latter. The *Bp*GH117 catalytic water is represented as a red nonbonded sphere

The structure of GH117B was determined to a resolution of 2.35 Å using *Bp*GH117 as a molecular replacement model (pdb entry: 4AK5^47^). Similar to other GH117 enzymes^45^, GH117B is a dimer comprised of two 5-bladed β-propeller monomers that are stabilized by interactions between the N terminus of one monomer interacting with the C terminus of the other monomer (Fig. 4a, Supplementary Fig. 5a). By soaking crystals of GH117B with N2, the structure of the enzyme was obtained in complex with its hydrolyzed products (2.4 Å). Clear electron density revealed a β-GAL residue in the +1 subsite and a α-AGH in the −1 subsite, the latter of which corresponds to the terminal residue on the non-reducing end of the substrate (Fig. 4b, Supplementary Fig. 5b). Similar to the *Bp*GH117 substrate complex, H392 from the Chain B monomer protruded into the active site of Chain A and interacts with the GAL product, indicating the conserved requirement of an intact dimer to generate a fully functional +1 subsite (Fig. 4b). Previous structures of GH117 enzymes have been reported in complex with non-hydrolyzed N2^47^ (Fig. 4c) and β-AHG^45^. The GH117B complex reported here, however, provides a unique snapshot of the enzyme interactions with both of the final monosaccharide products generated during agarose hydrolysis. This structure also revealed that there is no obvious steric impediment to the non-reducing ends of NAOS accessing the active site of GH117B, revealing how the enzyme converts NAOS to AOS, thereby generating substrates for GH2C (Fig. 2g).

**Fig. 5:**
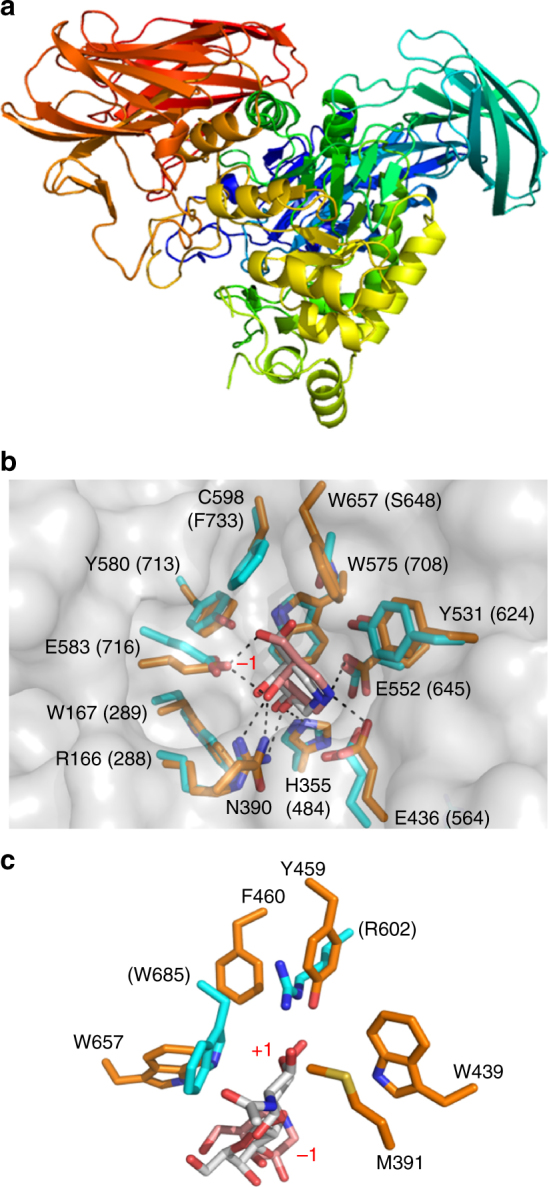
Molecular basis of the *exo-*acting β-1,4-d-agarooligosaccharide hydrolase, GH2C. **a** Structure of GH2C shown in cartoon format color ramped from N terminus (blue) to C terminus (red). **b** Surface representation (gray) of the GH2C catalytic pocket (orange) in complex with GIF (pink), overlaid with *S. pneumoniae* BgaA (cyan) in complex with GIF (gray) (pdb entry 4CU7^52^). Key GH2C residues are labeled, with corresponding BgaA residues in brackets. The −1 subsite is indicated and hydrogen bonds are represented by dashed lines. **c** Structural alignment of GH2C (orange) in complex with GIF (pink) (key residues labeled) with BgaA (cyan) in complex with *N*-acetyllactosamine (gray) (pdb entry 4CUC^52^). Subsites are indicated. Residues corresponding to BgaA are shown in brackets

To complete the comprehensive structural analysis of the *exo-*acting machinery within the *Bu* NP1 Ag-PUL, the apo-structure of GH2C was determined (2.5 Å; Fig. 5a). The *exo*-β-1,4-galactosidase displays an (α/β)_8_ barrel catalytic domain within a nest of Ig-like domains similar to other GH2 enzymes^51^; and the placement of catalytic residues within an active site pocket are characteristic of the GH2 *exo*-β-glycoside hydrolases. Soaking crystals of wild-type protein or crystals of an inactivated nucleophile mutant (E552Q mutation) with the substrate A3 failed to produce complexes. As an alternate strategy, crystals of the wild-type enzyme were soaked with the inhibitor galactoisofagomine (GIF), which produced interpretable electron density for the compound at the base of the active site pocket (2.4 Å; Supplementary Fig. 6). The orientation of GIF in the active site was very similar to that observed for a complex of the *Streptococcus pneumoniae* GH2 β-galactosidase (BgaA) with GIF and approximates the expected position of terminal β-linked GAL at the non-reducing end in the −1 subsite of the enzyme (Fig. 5b)^52^. The GAL binding subsite (i.e., −1 subsite) was closely conserved between the two enzymes and differs primarily in that S648 and F733 in BgaA are replaced with W657 and C598 in GH2C, respectively. However, comparison of GH2C to the complex of BgaA with unhydrolyzed *N*-acetyllactosamine indicated that the enzymes possess very different +1 subsite architectures (Fig. 5c). It is likely that this altered arrangement in the +1 subsite of GH2C allows the enzyme to specifically accommodate the β-linked AHG residue. Indeed, an amino acid sequence alignment of the agarose-active *Vibrio* sp. EJY3 GH2 β-galactosidase with GH2C indicates excellent conservation of the amino acids comprising the putative +1 subsite and supports the contention that this subsite is specifically contoured in both enzymes to recognize AHG (Supplementary Fig. 2b). These insights provide structural evidence of the agarose-tailored *exo*-β-d-galactosidase activity of GH2C.

### Growth of a non-agarolytic bacterium on GAL released from agarose

**Fig. 6:**
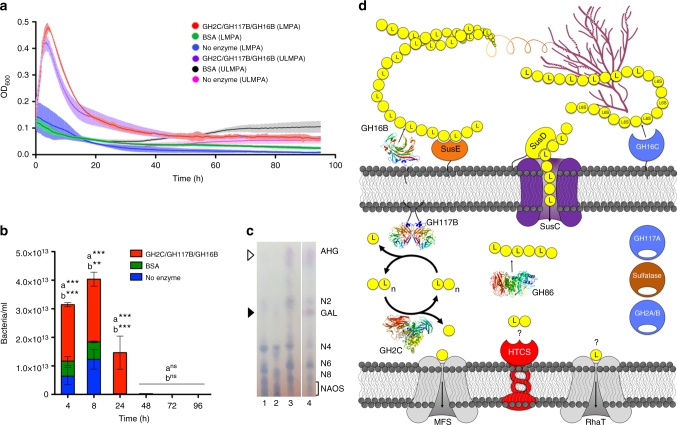
Role of agarolysis in selective growth of intestinal *Bu* and architecture of the Ag-PUL. **a** Growth of *Bu* ATCC 8492 grown on 1% (w/v) LMPA or ULPMA with growth medium supplemented with GH2C, GH117B, and GH16B. Error (s.d.) is represented by the thickness of the trace over three biological replicates, with six independent measurements per replicate. **b** Quantification of bacterial growth on LMPA was monitored by colony forming units (bacteria/ml). Vertical lines associated with histogram bars represent s.e.m. (*n* = 3); ^a^enzymes to BSA, ^b^enzymes to no enzyme; **p < 0.01, ***p < 0.001. **c** TLC analysis of the spent supernatant fractions following growth of agarolytic and non-agarolytic *Bu* strains on LMPA and ULMPA (representative replicates). Lane 1,* Bu* NP1 growth on LMPA, Lane 2, *Bu* NP1 growth on ULMPA, Lane 3*, Bu* ATCC 8492 on LMPA supplemented with GH2C, GH117B, and GH16B, and Lane 4, LMPA digestion control with GH16B, GH117B, and GH2C (no cell control). **d** Schematic model of the agarolytic pathway. Locations are based upon signal peptides listed in Supplementary Table 1. Initial depolymerization of agarose and porphyran occurs at the outer surface of *Bu* NP1 by the activity of GH16B and GH16C, respectively. The extracellular recruitment of agarose is facilitated by interactions with the SusE-like (NP1_9, orange) and SusD-like (NP1_10, yellow) proteins. Import of products occurs through the SusC-like transporter (NP1_11, purple). Within the periplasm, NAOS are further depolymerized to N2 by GH86; alternatively, NAOS can be saccharified by the *exo*-acting GH117B–GH2C cycle. Released GAL is imported into the cytoplasm through MFS proteins; AHG may have an alternative import pathway through the RhaT protein. Structurally characterized enzymes are shown in cartoon format color ramped from N terminus (blue) to C terminus (red), and predicted enzymes are displayed as spheres

To further examine the impact of Ag-PUL agarases acquired by *Bu* NP1, we attempted to augment the metabolic potential of the non-agarolytic bacterium *Bu* ATCC 8492 by adding purified recombinant (i.e., exogenous) agarases to cultures provided with agarose as a sole carbon source. Approximately 50% of the dry weight of agarose is GAL, a common nutrient for Bacteroidetes that is inaccessible to non-agarolytic microorganisms because of its refractory linkages with AHG. Thus, the release of GAL by the action of exogenous agarases should generate a product that can only then be utilized by non-agarolytic microorganisms. Using this rationale, the growth of *Bu* ATCC 8492 was successfully transformed to similar levels achieved by *Bu* NP1 (Fig. 1b) on both LMPA and ULPMA by adding GH16B, GH117B, and GH2C to the culture (Fig. 6a, b). In this manner, *Bu* NP1 agarases are able to generate equitable quantities of metabolizable products from both LMPA and ULMPA perhaps by digesting regions with similar structures (e.g., low modifications). Analysis of the spent supernatants revealed that the non-agarolytic strain *Bu* ATCC 8492 consumed free GAL from the culture media (Fig. 6c), further supporting that the monosaccharide subunits of agarose are utilized following their release from agarose by agarases.

### Architecture of the* Bu* NP1 agarolytic pathway

The archetypical PUL system contains outer membrane-bound, surface-exposed *endo-*acting hydrolytic enzymes that process large and/or chemically complex extracellular polysaccharides into smaller fragments^53^. These fragments are transported into the cell through SusC-like TonB-dependent transporters, a process that is typically facilitated by SusD-like, SusE-like, and other surface glycan binding proteins (e.g., SusF^54,55^). The remaining complement of enzymes is often trafficked into the periplasm^56^. This cellular organization ensures that the majority of substrate processing occurs within the periplasm, which is in proximity to sugar sensing domains of PUL transcriptional regulators and limits product loss to the environment. Typically, monosaccharides generated by periplasmic enzymes are transported into the cytoplasm for fermentation through major facilitator superfamily (MFS) systems^57^.

Informed by biochemical and structural insights into the function of the agarases within the Ag-PUL, we developed a model for the flow of agarose metabolism in *Bu* NP1 (Fig. 6d). In this regard, processing of the agarose and porphyran glycans is initiated by GH16B and GH16C, respectively, which are predicted to be displayed on the outer membrane surface (Supplementary Table 1). Products from these reactions are imported through the SusC/D/E-like transport system. Affinity gel electrophoresis determined that the SusD-like (NP1_10; *K*_d_ = 0.55 mM) and SusE-like (NP1_9; *K*_d_ = 0.037 mM) proteins bind agarose (Supplementary Fig. 7) with affinities that fall within ranges reported for surface glycan binding proteins from other PULs^55,58^. Interestingly, the SusE-like protein displayed weak affinity for native porphyran, which may reflect interactions with the agarose segments in this heterogeneous polysaccharide. Although porphyran can be cleaved by GH16C, it does not appear to be preferentially transported. This suggests that removal of porphyran may be a tangential process required for the efficient digestion and import of agarose. Transported NAOS are further depolymerized by GH86, and systematically saccharified by the *exo-*acting GH117B and GH2C enzymes. The presence of an upregulated RhaT homolog in the Ag-PUL (*np1*_25), which is a component of the cassette encoding the intracellular metabolic enzymes, suggests that there may be a dedicated system for l-sugar intracellular transport and that it may be selective for AHG. Energy harvest from the monosaccharide components of agarose is performed within the cell. GAL would be metabolized through glycolysis following epimerization by the Leloir pathway^40^. AHG is likely metabolized by the transferred metabolic cascade encoding the enzymes related to AHGD and AHDI (Supplementary Table 2).

### The selective role of agarolysis

A previous study defined that yeast mannan utilization in *Bacteroides* spp., is a “selfish process”^59^. This feeding strategy depends upon the complexity of the substrate^60–62^, and therefore each system should be considered independently. During selfish metabolism, initial products generated at the cell surface are rapidly funneled into the periplasm to prevent operational losses to other members of the local community. This contrasts what is seen for a distributive metabolism, commonly performed by “keystone” Gram-positive bacteria^63^, in which microorganisms collaborate at different stages of substrate depolymerization to release “public goods” to the microecosystem^62,63^.

Similar to yeast mannan, the biochemical evidence and proposed model of agarolysis presented here (Fig. 6d) suggest that utilization of agarose by *Bu* NP1 proceeds in a selfish manner. Following growth on agarose, the supernatants of the agarolytic *Bu* NP1 are depleted of N2, GAL, and AHG (Fig. 6c and Supplementary Fig. 8), highlighting that surface-exposed agarases (i.e., GH16B) do not generate NAOS smaller than N4, which is in agreement with the product profile of GH16B (Fig. 2d, f); and that monosaccharides are generated after transport, which is consistent with the *exo*-hydrolysis by GH117B and GH2C occurring within the periplasm (Supplementary Table 1). This cascade would prevent the release of free GAL to community members not endowed with functional agarases and ensure that only products (i.e., NAOS) with rare and potentially inaccessible linkages are generated. Accumulation of NAOS ≥4 in the media likely results from saturated transport kinetics if GH16B is generating products at a faster rate than they are imported through the SusC/D complex; indeed, agarose in the intestinal environment would likely be present in much smaller quantities. Alternatively, depletion of NAOS <N4 may also result from the presence of alternative transporters selective for N2, GAL, and AHG. The lack of accumulation of AHG in the media following growth of *Bu* NP1 on agarose substrates (Fig. 6d) supports a biological role for the predicted intracellular AHG metabolic pathway, as non-metabolized carbohydrates would otherwise be expected to be excreted from the cell, as has been observed previously^64^.

To investigate the ecological implications of agarolysis by *Bu* NP1, we attempted unsuccessfully to foster growth of *Bu* ATCC 8492, a non-agarolytic strain of *Bu*, on the spent supernatants of *Bu* NP1 following its growth on LMPA (Supplementary Fig. 8). The lack of cooperative nutrient utilization between these two closely related species is consistent with a selfish mechanism by *Bu* NP1. This observation, in combination with a lack of growth phenotype of *Bu* ATCC 8492 on intact agarose (Fig. 1b), suggested that utilization of carbohydrates locked with agarose polysaccharides and oligosaccharides is dependent on the genomic acquisition and deployment of functional agarases.

### The geographical distribution of algal galactan PULs in humans

The symbiotic metabolism of algal galactans by terrestrial animals, first observed in the porphyranolytic *Bp* DSM 17135 bacterium enriched in Japanese populations^31,32^, suggested that the distal gut microbiome is a dynamic community that responds to shifts in diet resulting from geography, culture, and/or lifestyle. The presence of complete pathways flanked by mobile elements indicates that the transfer of complete pathways within *Bacteroides* spp. is fluid and not simply the enrichment of established microbial specialists^29,31^.

**Fig. 7:**
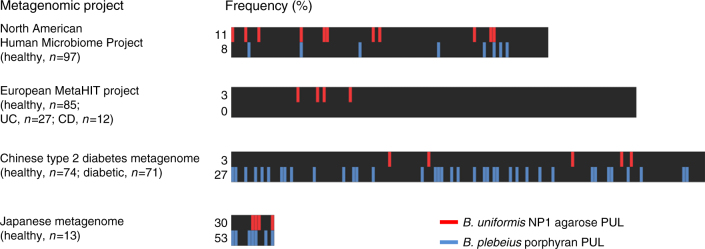
Abundance of algal galactan-specific PULs in human microbiomes. Four metagenomic data sets were individually queried by BLAST using the trimmed *Bu* Ag-PUL nucleotide sequence and the porphyran PUL sequence from *Bp*. Data were organized by metagenomic projects, the general information of which is listed on the left (UC ulcerative colitis, CD Crohn’s disease). Vertical lines representing the *Bu* NP1 Ag-PUL (red) and *Bp* porphyran PUL (blue) indicate the absence or presence of this PUL in a single individual. Black bars represent the total size of each metagenomics project, with the frequency of hits for each PUL shown to the left of each black bar

To further explore the distribution of algal galactan utilization within human-associated *Bacteroides* spp., four metagenomic data sets (*n* = 379 individuals) from regions with distinct geography, culture, and lifestyle (North America, Europe, China, and Japan; Fig. 7) were probed. When viewed globally, approximately 6% of the surveyed population harbors the *Bu* NP1 Ag-PUL, while 14% carries the *Bp* porphyran PUL, indicating that the utilization of dietary agarose might be rare among human beings. When analyzed for frequency within select datasets, however, it is apparent that differing cultures display distinct trends. In the Japanese metagenomes, nearly one in three individuals (30%) had microbiomes that tested positive for the Ag-PUL, whereas its prevalence was lower in Chinese (3%) and European (3%) metagenomes. This indicates that potential differences exist in dietary habits that may be influenced by historical traditions, proximity and accessibility to coastal feedstocks, and the global distribution of red algae habitats. Intriguingly, the higher levels of the Ag-PUL in North American metagenomes compared to European, and higher levels of the porphyran PUL in Japanese (53%) compared to Chinese (30%) metagenomes, suggest that complex anthropomorphic factors (e.g., immigration and culture) may be influencing the penetrance and distribution of algal galactan metabolism.

## Discussion

Biochemical work presented herein highlights that the growth of *Bu* NP1 on polymerized agarose is dependent on the expression of functional agarases. Our findings strengthen the emerging hypothesis that gain-of-function PUL transfer between Bacteroidetes occurs en bloc between donor and recipient species^29,31^, and underscore that rare nutrient utilization by intestinal bacteria is facilitated by the acquisition of enzymes that unlock inaccessible carbohydrate resources contained within unusual polysaccharide substrates.

Whether digestion of marine algal galactans by human intestinal bacteria is solely for the nutritional benefit of the microbe or if it provides evidence of dynamic, emergent mutualism in response to dietary change remains to be established. Intestinal *Bacteroides* spp. are well known for their pervasive role as “generalist” foragers^42,65^ and, correspondingly, they are enriched in enzymes devoted to the modification of structurally distinct polysaccharides^62^. The metabolic signatures of generalists suggest that there is a selective advantage in responding to a diverse glycan diet. This perceived advantage must be significant enough to ensure the maintenance of large segments of the genome that may remain dormant for generations. The origins of these pathways are more difficult to comprehend as selection drives the evolution of enzyme specificity (e.g., GH2, Supplementary Fig. 2) and assembly of complementary enzymes, transporters, and regulatory proteins into functional pathways (Fig. 1a)—all the while, nutrients that are more easily metabolizable may be readily available. For porphyran metabolism, it was hypothesized that the PULs arose within marine ecosystems before transfer to *Bp* DSM 17135, and after transfer, selective pressures on transformed intestinal bacteria were provided by dietary selection within a culture that consumed sufficient levels of porphyran in their diets^31,32^. In theory, dietary shifts would weaken or strengthen the selective pressures on such pathway evolution, and therefore targeting complex polysaccharide substrates that represent non-competitive nutritional opportunities may provide such fitness advantages to foraging bacteria. Regardless of the selective pressures guiding the emergence of algal polysaccharide metabolism within intestinal microecosystems, we provide support here that the en bloc transfer of the Ag-PUL results in gain-of-function metabolism for *Bu* NP1. Further research into the evolvability of PUL systems that target complex or uncommon polysaccharides, and the consideration of lifestyle factors, such as historical versus contemporary diets, geography, and culture, will help illuminate the emergence and sustainability of other intriguing metabolic pathways.

## Methods

### Materials

All reagents and chemicals were purchased from Sigma-Aldrich (St. Louis, MO) unless otherwise specified.

### Annotation of the Ag-PUL

The Ag-PUL was first analyzed for the presence of candidate GH117 and GH86 genes within the genome of *Bu* NP1 (available on the Broad Institute website: www.broadinstitute.org/) using the dbCAN website^36^. The genetic neighborhood surrounding identified candidate agarases were then systematically characterized using Artemis^38^. Identified ORFs were trimmed, exported, and annotated manually using dbCAN^36^ and BLASTp^37^. To determine the putative region of insertion of the Ag-PUL into the ancestral *Bu* chromosome, the non-agarolytic strain *Bu* ATCC 8492 was used as a sequenced referenced genome. The genomes of each bacterium were compared using Sequencher (Ann Arbor, MI). Contigs that aligned with 60% sequence similarity over a 40 kbp region were gathered for further analysis. After aligning the genomes, it was observed that the deposited genome of *Bu* ATCC 8492 was incomplete in this region and did not provide full sequence coverage. Therefore, the sequence region between contigs AAYH02000031.1 and AAYH02000048.1 was amplified using PCR primers *Bu*ATCC8492_AgPULFor2 (CACATATCCGGCAGCCTTACTTGCATTTCC) and *Bu*ATCC8492_AgRev3 (TCAACTATTGGTGGTTGCGTTGTCCTAACA) and sequenced. To determine the exact site of insertion, 50 kbp upstream and downstream of the insertion site in *Bu* ATCC 8492 and *Bu* NP1 was determined using webPRANK via EMBL^66^. Regions adjacent to expected insertion sites were then extracted from this sequence data and the 50 kbp regions were aligned.

### Growth profiling of *B. uniformis* on agarose substrates

All growth experiments were conducted at 37 °C in an anaerobic chamber (Coy Lab Products, Grass Lake, MI) in an atmosphere consisting of 85% N_2_, 10% CO_2_, and 5% H_2_. The type strains *Bu* NP1 and *Bu* ATCC 8492 were pre-grown in brain-heart infusion (BHI; Franklin Lakes, NJ) medium overnight. The cells were pelleted using a benchtop compact mini centrifuge (Mandel Scientific Company Inc., Guelph, ON) and washed with 2× minimal medium (MM) ^67^ containing 8.5 mM NH_4_SO_4_, 9.4 mM Na_2_CO_3_, 4.1 mM l-cysteine free base, 100 mM KH_2_PO_4_ pH 7.2, 1.4 μM FeSO_4_•7 H_2_O, 5 ng/ml vitamin B_12_, 1 μg/ml vitamin K_3_, 15.4 mM NaCl, 0.24 mM CaCl_2_, 98 μM MgCl_2_•6 H_2_O, 50 μM MnCl_2_•4 H_2_O, 42 μM CoCl_2_•6 H_2_O, and 1 μg/ml resazurin. The cells were resuspended in 2× MM supplemented with 2 μl/ml (v/v) histidine/hematin solution (1.9 μM hematin/200 μM l-histidine, prepared together as a 1000× solution) to a final OD_600_ ~ 0.2. Growth curves were performed in Falcon 300 μl flat-bottomed 96-well microtiter plates. Each well was loaded with 100 μl each of sterilized 1% (w/v) LMPA (IBI Scientific, Peosta, IA, IB70061), ULMPA (Sigma-Aldrich, A5030), and d-(+)-galactose (Sigma-Aldrich, G0750), as well as 100 μl of inoculum in technical triplicates to produce cultures with a final concentration of 1× MM and 0.5% (w/v) carbohydrate. For the sequential growth of *Bu* ATCC 8492 on *Bu* NP1 products, spent supernatants from *Bu* NP1 growth on 1% (w/v) LMPA (as above) were collected by resuspending the contents of each sample well in 100 μl distilled H_2_O, and removing the cells by centrifugation at 14,000 rpm for 5 min. The reaction was then stopped by boiling at 95 °C for 1 min, and 100 μl of the resulting products were mixed with 100 μl of *Bu* ATCC 8492 inoculum (as above). Wells containing 100 μl of bacterial suspension and 100 μl of water were used as negative controls as well as for buffer subtraction, and wells containing 180 μl BHI and 20 μl of the bacterial suspension were used as positive controls. Assay plates were sealed using clear Breathe-Easy gas-permeable polyurethane membranes (Sigma-Aldrich, Z280059) and loaded into a robotic plate stacking and handling device (BioTek, Winooski, VT) coupled to an Eon microplate reader (BioTek). Absorbance at 600 nm in each well was monitored every 10 min for 48 h. Data were processed using Gen5 software (BioTek), and displayed using GraphPad Prism.

### RT-PCR analysis of Ag-PUL induction

Three replicate cultures of *Bu* NP1 were pre-grown anaerobically in tryptone-yeast-glucose (TYG) broth overnight at 37 °C. The cultures were then washed in 2× MM with no carbon prior to the evaluation of growth in a single carbon source. MM supplemented with 1% (w/v) LMPA, ULMPA, or d-(+)-glucose were then inoculated with equal volume of suspended culture in Falcon 300 μl flat-bottomed 96-well microtiter plates. Assay plates were sealed with clear Breathe-Easy gas-permeable polyurethane membranes (Sigma-Aldrich, Z280059) and loaded onto Eon microplate reader (BioTek). The cultures were grown to mid-log phase prior to sampling and six technical replicate wells (200 μl each) from each carbohydrate treatment and each biological replicate were pooled for RNA extraction and subsequent RT-PCR analysis. Pooled cultures were placed in RNAprotect (Qiagen, Hilden, Germany, 76526) prior to purification with GeneJET RNA purification Kit (Thermo Fisher Scientific, K0731). RNA sample quality and quantities were verified using an Agilent Bioanalyzer and determined to be of good quality (RNA integrity number ≥ 8) prior to complementary DNA synthesis using QuantiTect Reverse Transcription kit (Qiagen, 205313). RT-PCR was performed in a 384-well plate with PerfeCTa®SYBR Green SuperMix (Quanta Biosciences, Beverly, MA). Data were normalized to glucose expression levels and compared to two reference target genes (signal recognition particle protein and 50S ribosomal protein L9), which were evaluated for expression stability using GeNorm^68^. Statistical analysis was conducted using qBase^plus^.

### Molecular cloning and protein purification

The gene fragments encoding predicted proteins from the Ag-PUL were amplified by PCR from *Bu* NP1 genomic DNA using specific primers to introduce 5’ *NheI* *or Nde*I and 3’ *Xho*I restriction sites (Supplementary Table 5). The genes were designed to lack predicted signal peptides and encode polypeptides with nucleotide boundaries as follows: GH2A, 145–2544; GH2B, 61–2526; GH2C, 76–2547; GH16A, 64–798; GH16B, 67–1029; GH16C, 72–1383; GH86, 61–1941; GH117A, 58–1209; GH117B, 76–1209; NP1_10 (SusD-like), 61–1779, and NP1_9 (SusE-like), 55–1560. Site-directed mutagenesis was used to create a catalytically inactive version of GH86 (referred to as GH86 E322Q). The E322Q mutation was introduced with the specific primers using the quick-change method (Supplementary Table 5) (Stratagene, San Diego, CA). The amplified products were cloned into pET28a via the engineered restriction sites using standard molecular biology procedures. The resulting gene fusions encoded an N-terminal His_6_ tag fused to the protein of interest by an intervening thrombin protease cleavage site. Bidirectional DNA sequencing was used to verify the fidelity of each construct.

Recombinant vectors were transformed into *Escherichia coli* BL21 Star (DE3) cells (Invitrogen, Carlsbad, CA) and proteins were produced on Luria-Bertani (LB) broth supplemented with kanamycin (50 μg/ml) as previously described^21^. Briefly, bacterial cells transformed with the appropriate expression plasmid were grown at 37 °C until the culture reached an optical density of 0.8–0.9 at 600 nm. Protein production was then induced by the addition of isopropyl-d-1-thiogalactopyranoside to a final concentration of 0.5 mM, and incubation was continued overnight at 16 °C with shaking at 170 rpm. Cells were harvested by centrifugation and disrupted by chemical lysis^44^ or by sonication using 1 s medium intensity sonic pulses for 2 min. Proteins were purified from the cleared cell lysate by Ni^2+^-immobilized metal affinity chromatography followed by size exclusion chromatography using a Sephacryl S-100 or S-200 column (GE Healthcare, Conroy, ON). Purified proteins, in 20 mM Tris-HCl pH 8.0, 0.5 M NaCl, were concentrated using a stirred cell ultrafiltration device with a 10,000 Da molecular weight cut-off membrane (Millipore, Billerica, MA). Protein concentration was determined by measuring the absorbance at 280 nm and using the following calculated molar extinction coefficients: GH2A: 170,225 M^−1^ cm^−1^; GH2B: 211,455 M^−1^ cm^−1^; GH2C: 211,830 M^−1^ m^−1^; GH16A: 86,525 M^−1^ cm^−1^; GH16B: 844,65 M^−1^ cm^−1^; GH16C: 90,230 M^−1^ cm^−1^; GH86: 99,295 M^−1^ cm^−1^; GH117A: 87,125 M^−1^ cm^−1^; GH117B: 117,020 M^−1^ cm^−1^; NP1_9 (SusE-like): 72,205 M^−1^ cm^−1^; and NP1_10 (SusD-like): 119,220 M^−1^ cm^−1^ ^69^.

### Agarase assays

Enzymatic activities toward algal galactans were detected using reducing sugar assays. Enzymes at a concentration of 2 μM in Tris 20 mM pH 8.0 containing 0.5 M NaCl were incubated at 37 °C for 2 h in the presence of 1% agarose, raw and enriched (see below) porphyran, or κ-carrageenan. Working reagent containing reagent A (10 g of para-hydroxy benzoic acid hydrazide in 5% hydrochloric acid) and reagent B (trisodium citrate 12.45 mg/ml and calcium chloride 1.1 mg/ml in sodium hydroxide at 20 mg/ml) in a 1:9 v/v ratio was added to the reaction mixture, maintained at 100 °C for 10 min, and absorbance read at 410 nm. GAL at concentrations ranging from 0 to 20 mM was used as a standard.

Enriched porphyran was prepared from dried Nori. Briefly 50 g of *g*round dried Nori was resuspended into 1.2 L of distilled water and boiled for 5.5 h. After centrifugation (30 min, 8000 rpm) and filtration of the supernatant, raw porphyran was precipitated by addition of acetone (with a ratio supernatant:acetone of 1:4 v:v). Precipitated raw porphyran was then harvested by centrifugation (30 min, 8000 rpm), resuspended in clean water, and lyophilized. Raw porphyran was further purified into enriched porphyran by digesting agarose contaminants using known agarases. In brief, *Saccharophagus degradans* GH50 (540 μM)^21^, *Bacteroides plebeius* GH16A (470 μM), and GH117 (410 μM) were incubated over 3 days at 37 °C with shaking (90 rpm) in the presence of 1 g of raw porphyran, which results in the degradation of agarose polysaccharides into monosaccharides. After centrifugation (20 min, 8000 rpm), the digested raw porphyran was dialyzed for 2 days against clean water to remove monosaccharides. The enriched porphyran was then lyophilized. To be used as a control, raw porphyran was treated in parallel in the absence of agarases.

Product profiling was performed using TLC and FACE. TLC reactions were prepared using 0.5 μM of enzyme in 20 mM Tris pH 8.0 supplemented with 0.5 M NaCl on a bed of 0.8% (w/v) agarose (Invitrogen, 16500-500) at 37 °C for 24 h or using 2 μM of enzyme in 20 mM Tris pH 8.0, 0.5 M NaCl containing 2.5 mM of agarotriose (A3, Aglyco, Bejing, China) at 37 °C for 2 h. The reaction samples were then resolved by TLC with 1-butanol/acetic acid/distilled water (2:1:1, v/v/v) running buffer and visualized with one part 0.2% (w/v ethanol) 1,3-dihydroxynaphthalene (Sigma-Aldrich, N6250) to two parts 3.75:1 ethanol/sulfuric acid solution, followed by heating at 110 °C for 2–5 min. Samples were compared to standards of GAL, N2, A3, N4, N6, and N8 (NAOS were obtained as previously described^21^). The digestion of N2 with GH117B was performed by incubating 0.5 μM GH117B with 0.5 mg/ml N2 in 20 mM Tris pH 8.0, 0.5 M NaCl at 37 °C for 24 h. The FACE method was adapted from Jackson.^70^. The reactions consist of 2.5 μM of enzyme and agarose, porphyran, or κ-carrageenan at 0.5–1% in Tris 20 mM pH 8.0 containing 0.5 M NaCl, incubated overnight at 37 °C. The reaction products were then labeled overnight using 8-aminonaphthalene-1,3,6-trisulfonic acid disodium salt (ANTS, 0.02 M in acetic acid/water 3:17 v/v) and sodium cyanoborohydride (0.1 M in dimethyl sulfoxide). Approximately 0.2 μg of ANTS-labeled product were loaded onto a 35% polyacrylamide (19:1) gel with a 10% stacking gel and electrophoresed 30 min at 100 V followed by 1 h at 300 V at 4 °C in native running buffer (25 mM Tris-HCl, 0.2 M glycine). Gels were immediately visualized and imaged under ultraviolet light.

### Crystallization and structure solution of agarases

Crystallization experiments were performed using the vapor diffusion method at 18 °C with sitting drops for screening and hanging drops for optimization. For data collection, single crystals were flash-cooled with liquid nitrogen in crystallization solution supplemented with a cryoprotectant optimized for each crystal form as given below. Diffraction data were collected either on beamline 08B1–1 at the Canadian Light Source (CLS, Saskatoon, SK) or beamline BL7-1 at Stanford Synchrotron Radiation Lightsource (SSRL, Stanford, CA). All diffraction data were processed using MOSFLM^71^ and SCALA^72^. Data collection and processing statistics are shown in Supplementary Tables 7 and 8. All the structures were initially solved by molecular replacement using PHASER^73^. Then, a combination of automatic model building with BUCCANEER^74^, manual building with COOT^75^, and refinement with REFMAC^76^ was used. The addition of water molecules was performed in COOT with FINDWATERS and manually checked after refinement. In all data sets, refinement procedures were monitored by flagging 5% of all observation as “free”^77^. Model validation was performed with MOLPROBITY^67,78^. The models obtained were finally represented using the program PyMOL (PyMOL Molecular Graphics System). Modeling statistics are shown in Supplementary Tables 7 and 8.

Crystals of GH2C (25 mg/ml) were obtained in 0.2 M magnesium sulfate, 0.1 M Tris-HCl pH 8.5, 20% (w/v) polyethylene glycol (PEG) 3350. Crystals were cryoprotected in crystallization solution containing 20% (v/v) ethylene glycol. The structure was solved by molecular replacement using the structure of BgaA from *S. pneumoniae* (pdb entry: 4CU6^52^) as a model. Crystals, obtained in 0.2 M ammonium citrate, 20–24% (w/v) PEG 3350, were also soaked with an excess of GIF for 1 h prior to cryoprotection in 25% (v/v) ethylene glycol.

Crystals of GH16B (25 mg/ml) were grown in 0.2 M sodium chloride, 0.1 M imidazole-HCl, pH 8.0, 0.4 M NaH_2_PO_4_/1.6 M K_2_HPO_4_. Crystals were cryoprotected in crystallization solution containing 25% (v/v) ethylene glycol. The structure of GH16B from *B. plebeius* (pdb entry: 4AWD;^31^) was used as a model for molecular replacement.

Crystals of GH86 (28 mg/ml) were obtained in 0.16 M CaOAc, 0.08 M sodium cacodylate (C_2_H_7_AsO_2_), pH 6.5, 14.4 % (w/v) PEG 3350, and 20% (v/v) glycerol. Ethylene glycol (25% v/v) was used to supplement the crystallization solution for cryoprotection. Crystals, obtained in 0.2 M ammonium sulfate, 0.1 M Bis-Tris pH 5.5, and 21% (w/v) PEG 3350, were also soaked with an excess of N8 for 30 min prior to cryoprotection. The structures were solved using the structure of *B. plebeius* GH86A (pdb entry: 4AW7^31^) as a model for molecular replacement.

Crystals of GH86 E322Q (40 mg/ml) were obtained in the presence of 0.16 M CaOAc, 0.1 M C_2_H_7_AsO_2_, pH 6.2, 17.5% (w/v) PEG 8000, and 5% (v/v) glycerol. These crystals were soaked for 30 min in the crystallization solution containing an excess of N8 prior to freezing using 25 and 20% (v/v) ethylene glycol in crystallization solution as a cryoprotectant. These structures were solved by molecular replacement as described above.

Crystals of GH117B (7 mg/ml) were grown in 0.1–0.18 M LiSO_4_, 0.1 M Tris-HCl, pH 8.5, 25–30% (w/v) PEG 4000. Some crystals were cryoprotected directly in the crystallization solution supplemented with 20% (v/v) ethylene glycol and some were soaked with an excess of N2 for 30 min prior to cryoprotection. These structures were solved by molecular replacement using the *B. plebeius* GH117 structure (pdb entry: 4AK5^47^).

### Growth of *Bacteroides* spp. cultures using exogenous agarases

All growth experiments were conducted in an atmosphere consisting of 85% N_2_, 10% CO_2_, and 5% H_2_ at 37 °C unless otherwise noted. The *Bu* ATCC 8492 type strain was pre-grown in triplicate in chopped meat broth medium overnight. The cells were pelleted by centrifugation and washed once with 2× MM (see above) and resuspended in 2× MM supplemented with 2 μl/ml (v/v) histidine/hematin solution to a final OD_600_ ~ 0.2. Growth curves were performed in Falcon 300 μl flat-bottomed 96-well microtiter plates. Each well was loaded with 100 μl of 1% (w/v) LMPA or ULMPA and 100 μl of the bacterial suspension supplemented with 20 mM Tris-HCl pH 8.0 and either 1 μM each GH2C, GH117B, and GH16B, mass equivalent of bovine serum albumin (BSA) or water. Assay plates were sealed using clear Breathe-Easy gas-permeable polyurethane membranes (Sigma-Aldrich, Z280059) and loaded into a robotic plate handling device (BioTek) coupled to an Eon microplate reader (BioTek). Absorbance at 600 nm in each well was monitored every 10 min for 96 h. Data were processed using Gen5 software (BioTek) and Microsoft Excel, and displayed using GraphPad Prism. Post-growth supernatants were collected by resuspending the contents of each sample well in 100 μl distilled H_2_O, and removing the cells by centrifugation at 14,000 rpm for 5 min. The reaction was then stopped by boiling at 95 °C for 1 min. The reaction samples were resolved by TLC with 1-butanol/acetic acid/distilled water (2:1:1, v/v/v) running buffer and visualized with one part 0.2% (w/v ethanol) 1,3-dihydroxynaphthalene (Sigma-Aldrich, N6250) to two parts 3.75:1 ethanol/sulfuric acid solution, followed by heating at 110 °C for 2–5 min. To measure viable cells, the culture conditions described above were scaled up to 8 ml final volume comprised of 4 ml 1% LMPA and 4 ml of the bacterial suspension supplemented with 20 mM Tris-HCl, pH 8.0, and either 1 μM each GH2C, GH117B, and GH16B, mass equivalent of BSA, or water. Cells were maintained in the anaerobic atmosphere in 15 ml conical tubes for 96 h. Cultures were sampled at 4, 8, 24, 48, 72, and 96 h of growth and diluted in 1 ml of chopped meat broth in a 10-fold dilution series. Then, 25 µl from each dilution were spread in duplicate onto BHI agar supplemented with gentamycin (200 μg/ml), cultures were maintained in the anaerobic atmosphere for 72 h, and colony forming units were enumerated at the dilution yielding 15–300 colonies per culture. The mean of the three observations was calculated.

### Analysis of metagenomic data sets

Human metagenomic data sets were searched by BLAST for the presence of an agarose PUL nucleotide sequence from *Bu* NP1 (51.367 kb) and a porphyran PUL nucleotide sequence from *B. plebeius* (54.476 kb). These probe sequences were first searched against the NCBI Refseq genome database to identify BLAST hits to other sequenced genomes that do not contain an agarose PUL using megablast algorithm with default settings. Aligned regions from hits with E-values <1e−20 and nucleotide sequence identities >80% or >70% were removed from the agarose PUL or the porphyran PUL probe, respectively. The resulting trimmed probe sequences were used in subsequent BLAST searches using either BlastStation local 64 software or on IMG website. A metagenome was positive for a PUL if it returned two or more hits with ≥100 bp in length, ≥90% identity, and E-value ≤1 e−20. For metagenomic data sets with large contigs, the single hits that were ≥10 kb with the same identity and E-value cut-offs as listed above were also considered positive.

### Data availability

Accession codes for the crystal structures are available on the Protein Data Bank (PDB) as follows: GH2C (apo: 5T9A; GIF complex: 5T9G), GH117B (apo: 5T9X; N2 complex: 5TA9), GH16B (apo: 5TA1), and GH86 (apo: 5TA7; E322Q N8 complex: 5TA0). The data that support the findings of this study are available from the corresponding authors upon request.

## Electronic supplementary material

Supplementary Information
