# Supplementary material for: Molecular basis of an agarose metabolic pathway acquired by a human intestinal symbiont
Source: Nat Commun. 2018 Mar 13;9:1043. doi: 10.1038/s41467-018-03366-x (PMC5849685; doi:10.1038/s41467-018-03366-x)
Supplement: Supplementary file 1 — Supplementary Information [file 41467_2018_3366_MOESM1_ESM.pdf]

Supplementary Information

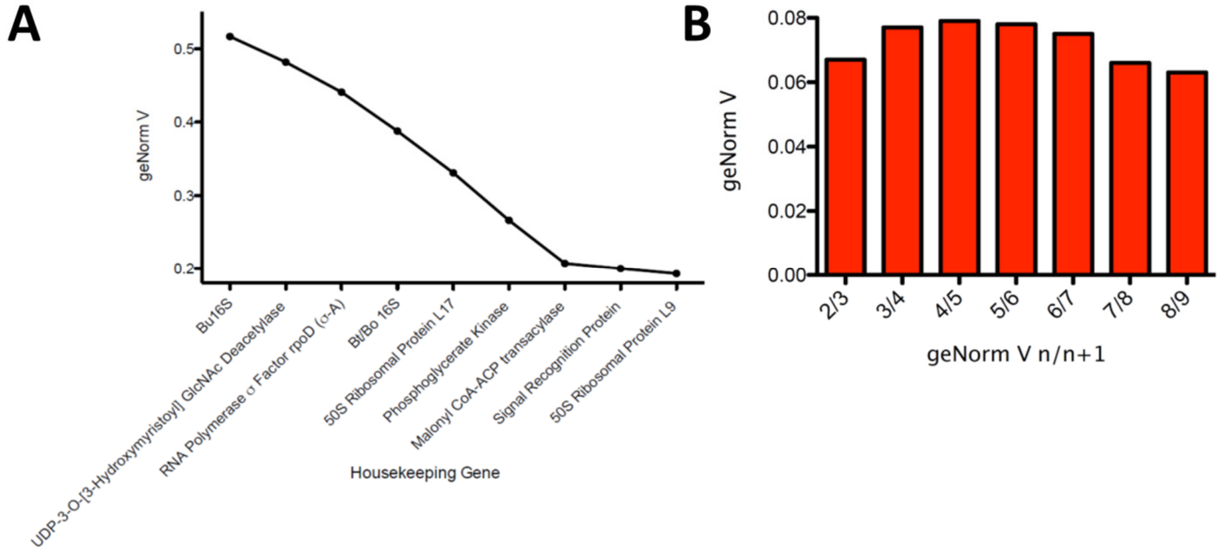

Supplementary Figure 1. Putative reference gene stability evaluation results using geNorm. (**a**) The putative housekeeping genes are ranked from most unstable (left, high M value, *Bu*16S) to the most stable (right, low M value, 50S Ribosomal Protein L9). geNorm V is < 0.15 when comparing the normalization factors based on using the two or three most stable reference genes. (**b**) Optimal normalization factors were calculated as the geometric mean of reference targets SRP and 50S_RP_L9 (23). Using these two genes as the reference targets resulted in a very high reference target stability (average geNorm M ≤ 0.2).

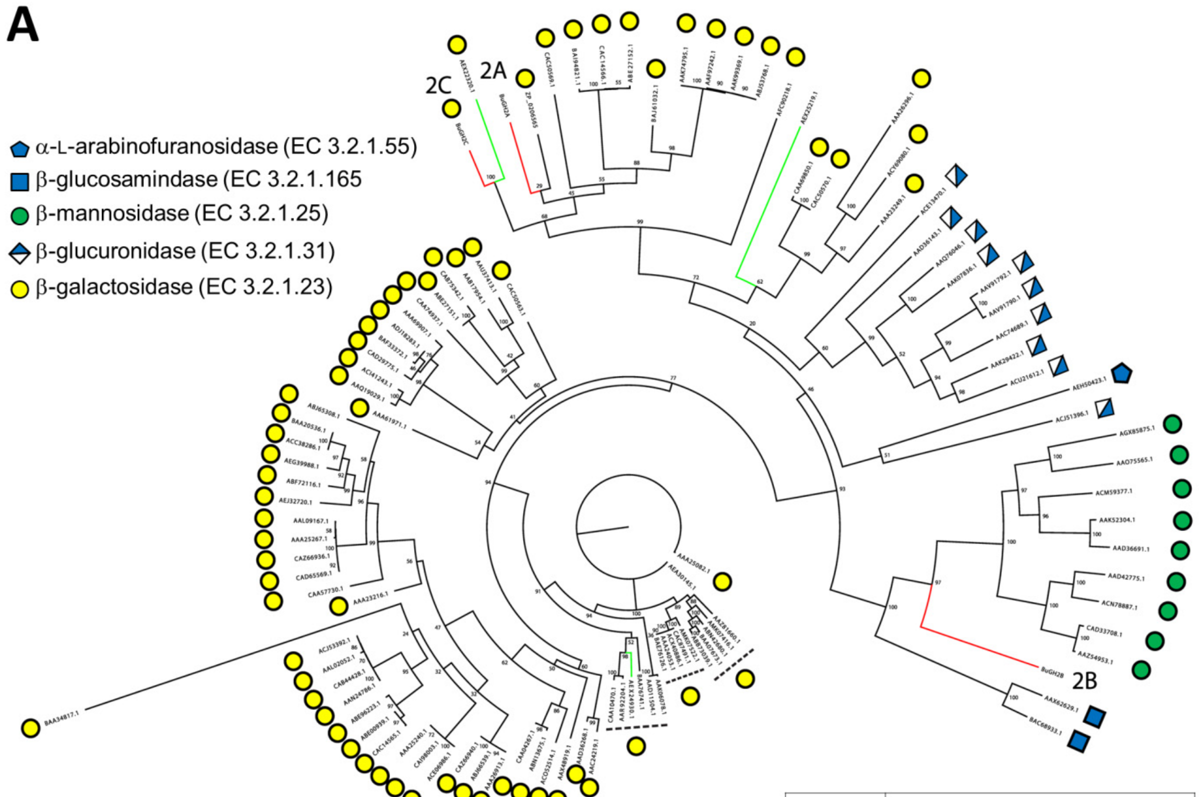

ENZYME	GH2B	GH2A	VejABG	GH2C
GH2B	100.0	20.9	17.9	20.2
GH2A	-	100.0	29.8	37.1
VejABG	-	-	100.0	42.7
GH2C	-	-	-	100.0

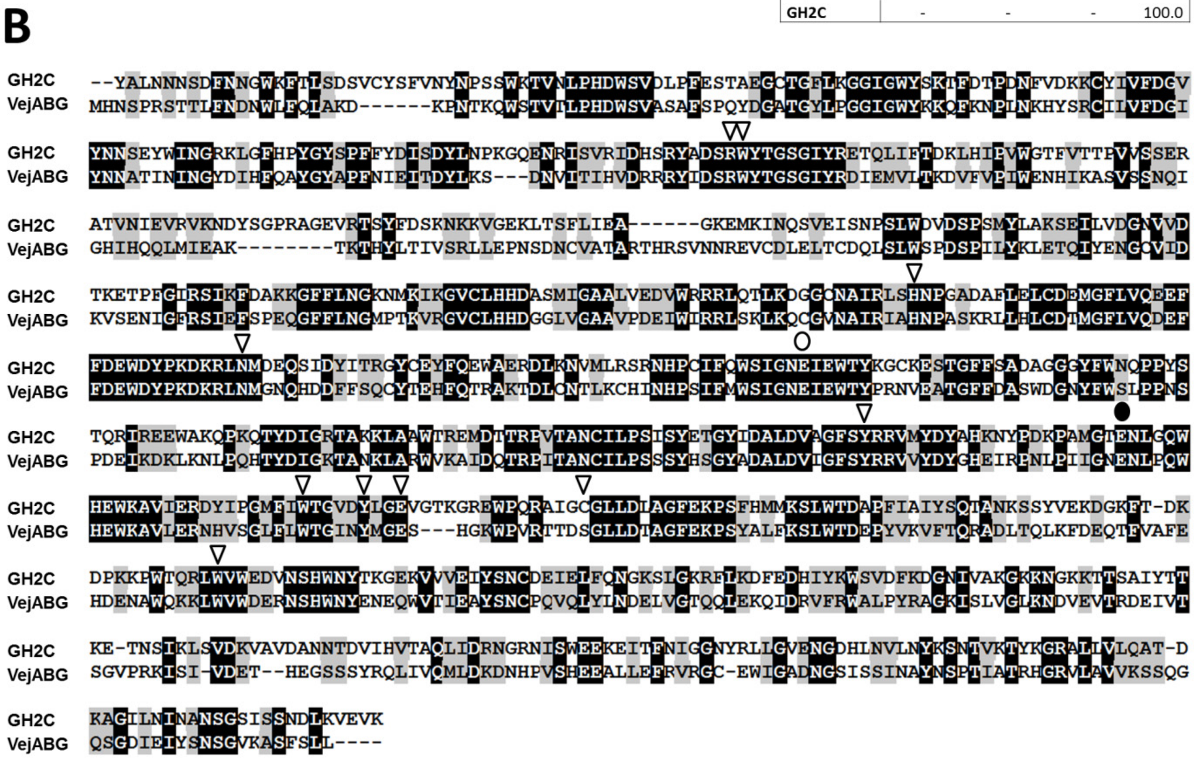

Supplementary Figure 2: Phylogeny of characterized GH2 enzymes. (a) Sequences were extracted from the CAZy database (17), trimmed to their GH2 catalytic module using dbCAN (18), and aligned using MUSCLE (24). The phylogenetic tree was generated using RAxML (20) and FigTree (http://tree.bio.ed.ac.uk/software/figtree/). Ascribed activities for GH2 include β-glucosamindase (EC 3.2.1.165; blue square), β-mannosidase (EC 3.2.1.25; green circle), β-glucuronidase (EC 3.2.1.31; blue/white diamond), β-galactosidase (EC 3.2.1.23; yellow circle), and α-L-arabinofuranosidase (EC 3.2.1.55; blue pentagon). Lineage of GH2s from *Vibrio* sp. EJY3 (25) are shown with green lines; and GH2s from *Bu* NP1 with red lines. Sequence identities between the GH2s from *Bu* NP1 and the GH2 from *Vibrio* sp. EJY3, VejABG, are shown as percentages. **(b)** Alignment of GH2C and VejABG. Residues comprising the putative +1 subsite in GH2C are denoted using white triangles. The catalytic acid/base (E436) and nucleophile (E552) are indicated using black triangles.

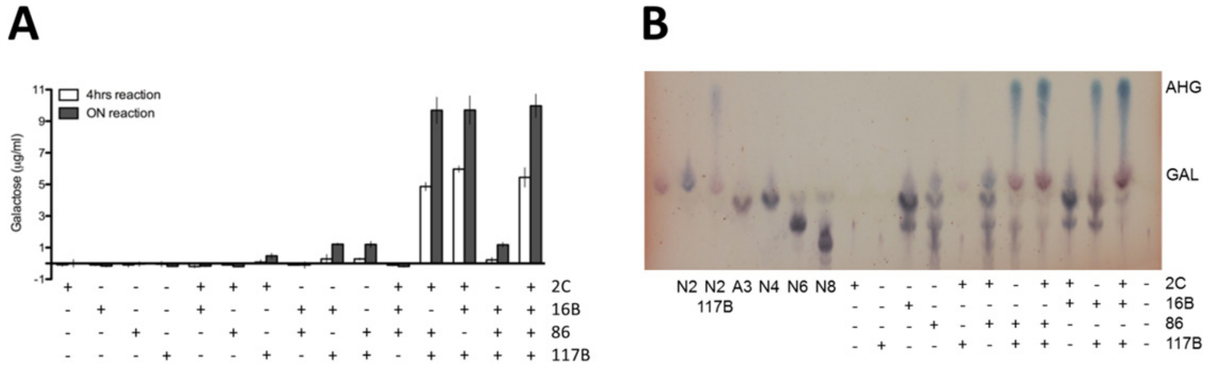

Supplementary Figure 3: Production of GAL an AHG by *Bu* NP1 Ag-PUL agarases. (**a**) Combinatorial cocktail digestions of agarose using GH86, GH16B, GH2C, and GH117B were performed and incubated for 4 hours or overnight. GAL was quantified in the resulting supernatants using a reducing sugar assay. Error (s.d.) is represented by error bars over four replicates. (**b**) TLC analysis of the complete factorial of GH86, GH16B, GH2C, and GH117B cocktail agarose digestions.

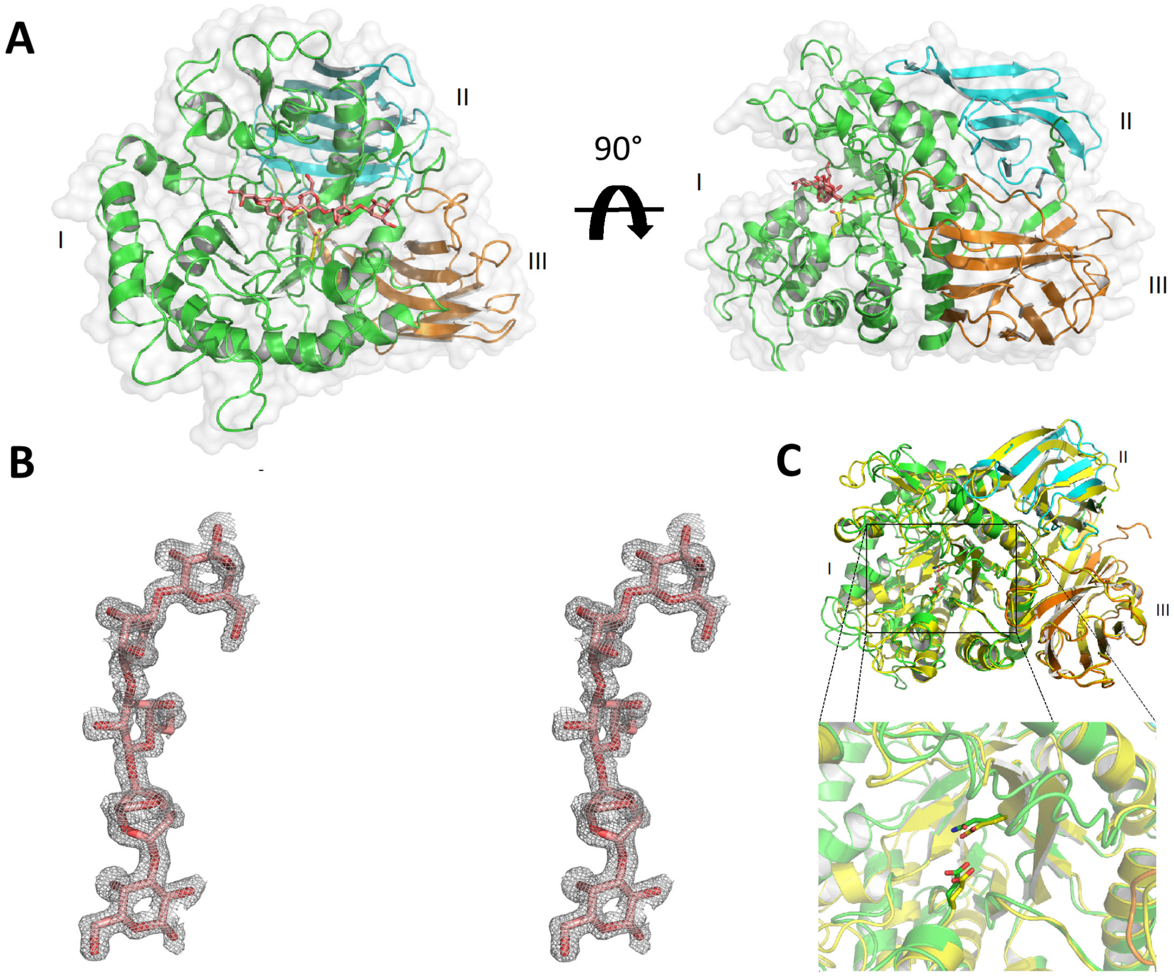

Supplementary Figure 4: Structural analysis of GH86 from the *Bu* NP1 Ag-PUL. (**a**) Structure of the inactive E322Q mutant of GH86 in complex with N8 (pink). Domains are indicated by Roman numerals. (**b**) Despite crystal soaks with excess N8, electron density allowed for the modelling of agaropentaose (A5) with a maximum likelihood^σ_a weighted $2F_{obs}$-F_{calc} electron density map contoured at 0.35 electrons^$Å^3$. (**c**) Structural alignment of *Bu*GH86 (green, cyan, orange) with *Bp*GH86A [yellow, pdb entry: 4AW7, (12)], with structurally conserved catalytic residues shown in stick format (inset). Domains are indicated by Roman numerals.

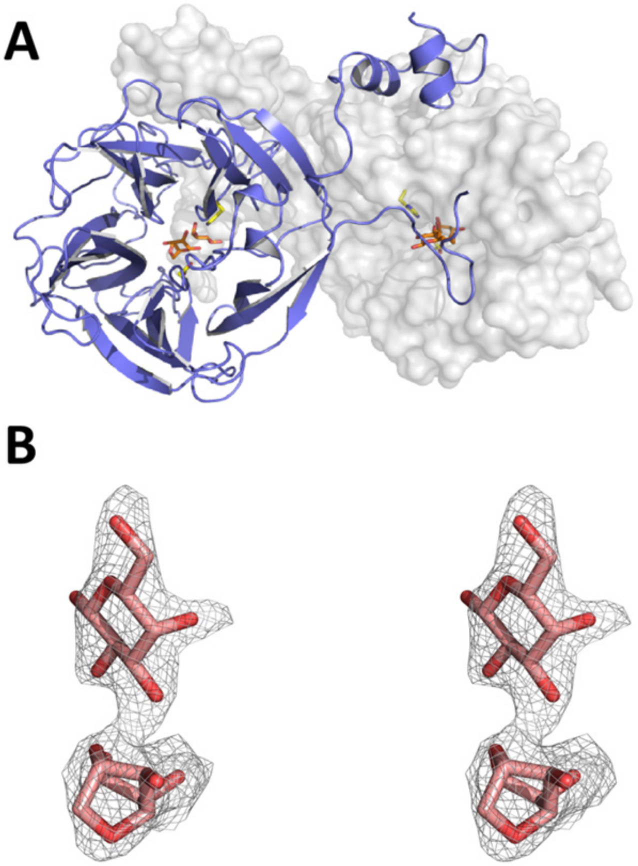

Supplementary Figure 5: Structural features of GH117B from the *Bu* NP1 Ag-PUL. (**a**) Chain A (cartoon, blue) and Chain B (surface, grey) of GH117B in complex with GAL and AHG (sticks, pink). (**b**) Electron density in the active site of GH117B was sufficient to model GAL and AHG with a maximum likelihood^σ_a weighted $2F_{obs}$-F_{calc} electron density map contoured at 0.25 electrons^Å^3.

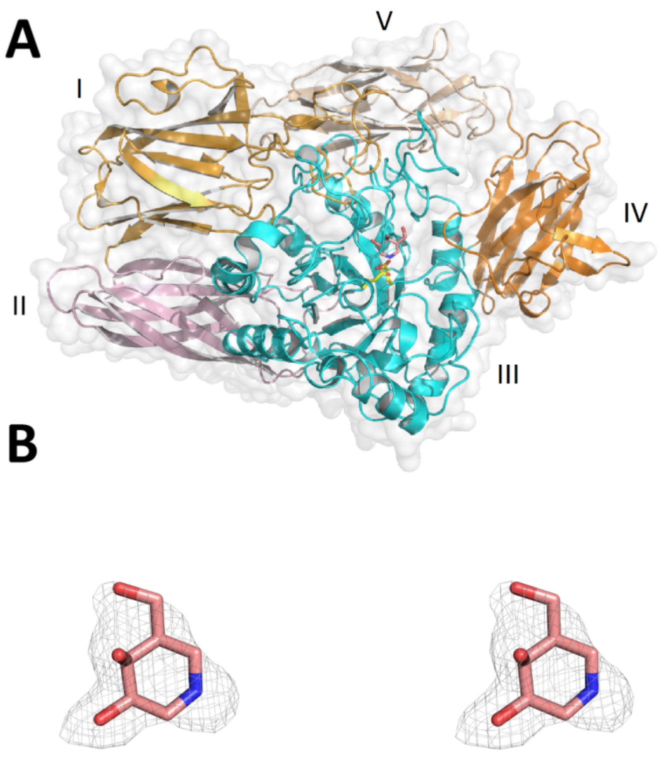

Supplementary Figure 6: Structure of GH2C in complex with the inhibitor galactoisofagomine (GIF). (**a**) Cartoon representation of the catalytic region comprising the catalytic domain (III, cyan), surrounded by four IG-like domains (I, II, IV, and V). The bound GIF molecule is shown in pink and the protein surface in transparent grey. (**b**) Electron density in the active site of GH2C was sufficient to model GIF with a maximum likelihood^σ_a weighted $2F_{obs}$-F_{calc}electron density map contoured at 0.27 electrons^Å^3.

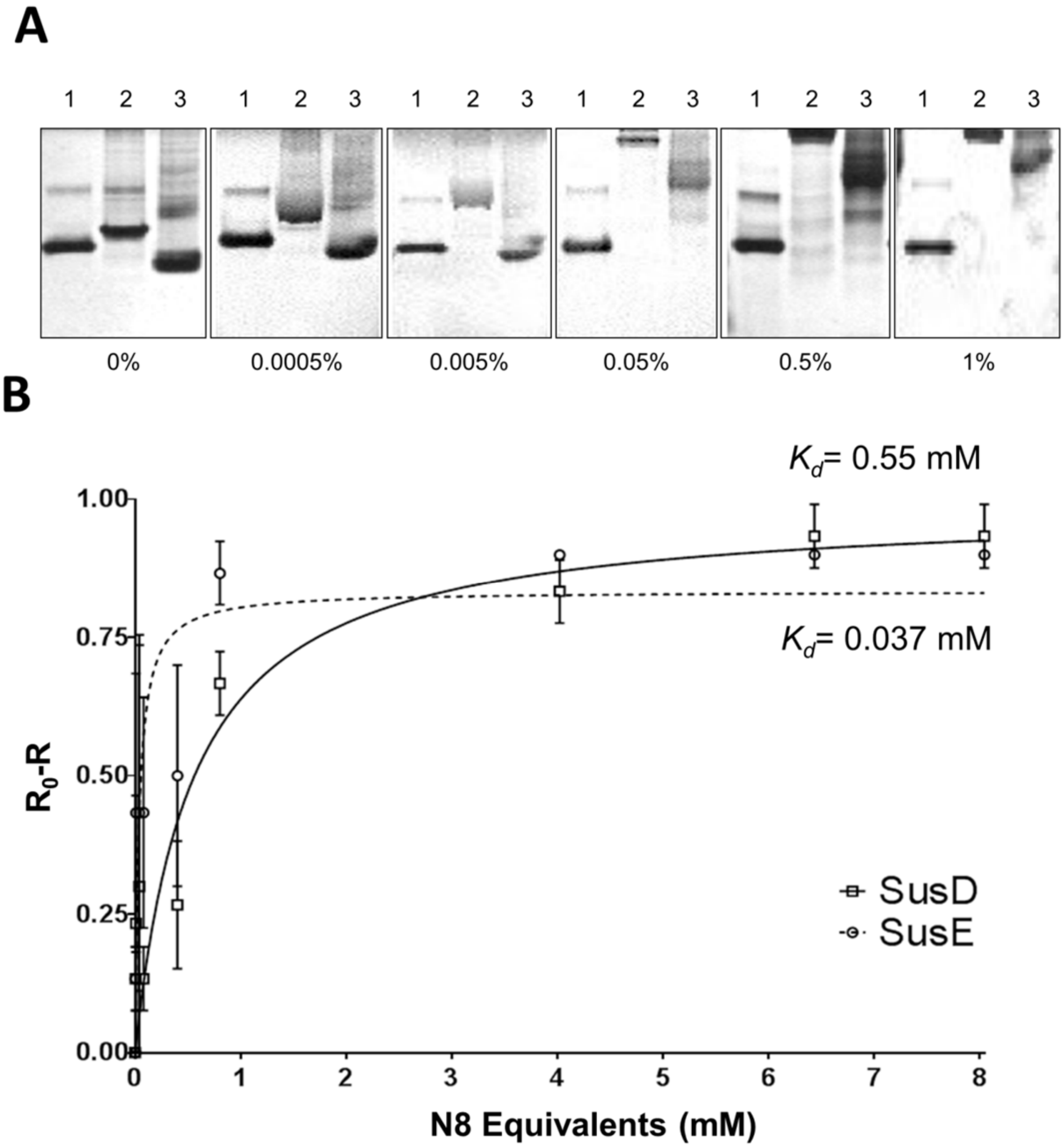

Supplementary Figure 7: Affinity gel electrophoresis of recombinant SusD- (NP1_10) and SusE-like (NP1_9) proteins. (**a**) Quantitative AGE using stepwise gradient of agarose-containing native gels, showing the relative mobilities for a BSA negative control (lane 1), SusE- (lane 2) and SusD-like (lane 3) proteins. (**b**) Normal relative mobility of SusD-like and SusE-like proteins plotted against N8 equivalents. Binding constants were fit to a one-site binding model and

calculated using GraphPad software. Error (s.d.) is represented by vertical error bars over three replicates.

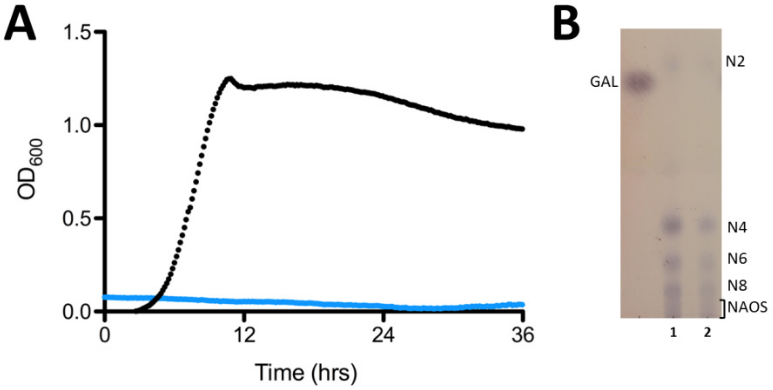

Supplementary Figure 8: Growth of *Bu* ATCC 8492 on spent supernatants from growth of BuNP1 on LMPA. (**a**) Growth curves of agarolytic *Bu* ATCC 8492 grown on GAL (black) and the spent supernatants of *Bu* NP1 following growth on LMPA (blue). The experiment was replicated three times, with four to eight observations per replicate; error (s.d.) is represented by trace thickness. (**b**) TLC analysis of the spent supernatants of *Bu* NP1 (lane 1) following growth on LMPA, and *Bu* ATCC 8492 following growth on the *Bu* NP1 spent supernatants (lane 2).

Supplementary Table 1: Annotation of the Agarolytic PUL from *B. uniformis* NP1

Gene	Predicted Protein	LipoP prediction[1]	Function[2]
*np1*_1	Cyclase	Cytoplasmic	Unknown
*np1*_2	DUF2582	Cytoplasmic	Unknown
*np1*_3	Unknown	SPI	Unknown
*np1*_4	BuAs1A-like (sulfatase)	SPII (2+=G, OM-CM)[2]	Sulfatase
*np1*_5	BuGH117A	SPI	Agarase/Porphyranase
*np1*_6	BuGH16A	SPI	Agarase/Porphyranase
*np1*_7	BUGH86	SPI	β-agarase*
*np1*_8	BuGH16B	SPII (2+=S, OM)	β-agarase*
*np1*_9	SusE-like	SPII (2+=Q, OM)	CBM-Containing, Transport
*np1*_10	SusD-like	SPII (2+=E, OM)	CBM-Containing, Transport
*np1*_11	SusC-like	ND	Outer Membrane Transport
*np1*_12	BuAs1A-like (sulfatase)	Cytoplasmic	Sulfatase
*np1*_13	Transferase/Transposase	Cytoplasmic	Chromosome Integration
*np1*_14	DDE_1	Cytoplasmic	Transposase
*np1*_15	Transferase/Transposase	Cytoplasmic	Chromosome Integration
*np1*_16	DDE_3	Cytoplasmic	Transposase
*np1*_17	HTCS	ND	Transcriptional Regulation
*np1*_18	Unknown	Cytoplasmic	Unknown
*np1*_19	BuAs1A-like	SPI	Sulfatase
*np1*_20	GH16C	SPII (2+=N, OM)	β-Porphyranase*
*np1*_21	GH29	SPII (2+=N, OM)	L-Galactosidase
*np1*_22	GH2A	Cytoplasmic	β-Galactosidase, Porphyranase*
*np1*_23	Unknown	Cytoplasmic	Unknown
*np1*_24	KdgK-like	Cytoplasmic	KDG Kinase
*np1*_25	RhaT-like	ND	Sugar Importer
*np1*_26	GalM-like	Cytoplasmic	Galactose Mutarotase
*np1*_27	MR-like	Cytoplasmic	Epimerase/Dehydratase/Decarboxylase
*np1*_28	AldA-like	Cytoplasmic	Aldehyde Dehydrogenase
*np1*_29	KduD	Cytoplasmic	KDG Oxidoreductase
*np1*_30	MDR-like	Cytoplasmic	Alcohol Dehydrogenase
*np1*_31	GH2B	SPI	β-mannosidase
*np1*_32	GH117B	SPII (2+=V, OM)	3,6-Anhdryo-L-Galactosidase*
*np1*_33	GH2C	SPI	β-Galactosidase, Agarase*

[1](26)
[2]Characterized enzymes are indicated with an asterisk. See Supplementary Table 8 for more detail.
[3](27)

Supplementary Table 2: Distance identity tree of metabolic enzymes with the AHG pathway from *Vibrio* sp. VEJ3[1]

Protein	AHGD	NP1_24	NP1_25	NP1_26	NP1_27	NP1_28	NP1_29	NP1_30
AHGD[2]	100.0	18.0	19.0	17.6	11.7	64.6	14.8	13.2
NP1_24	-	100.0	12.7	12.6	9.6	17.9	8.3	10.3
NP1_25	-	-	100.0	15.3	13.7	19.3	15.2	15.1
NP1_26	-	-	-	100.0	11.3	16.3	13.2	11.2
NP1_27	-	-	-	-	100.0	14.4	22.9	21.5
NP1_28	-	-	-	-	-	100.0	13.3	13.6
NP1_29	-	-	-	-	-	-	100.0	16.7
NP1_30	-	-	-	-	-	-	-	100.0
Protein	AHGI	NP1_24	NP1_25	NP1_26	NP1_27	NP1_28	NP1_29	NP1_30
AHGI[3]	100.00	15.70	13.30	10.64	72.10	16.13	20.00	16.37
NP1_24	-	100.00	12.50	15.58	14.05	15.85	9.76	18.14
NP1_25	-	-	100.00	22.86	14.22	15.69	12.02	14.65
NP1_26	-	-	-	100.00	10.04	15.16	5.81	11.49
NP1_27	-	-	-	-	100.00	14.29	22.18	17.80
NP1_28	-	-	-	-	-	100.00	13.36	19.62
NP1_29	-	-	-	-	-	-	100.00	12.89
NP1_30	-	-	-	-	-	-	-	100.00

[1](28).

[2]AHGD (VEJY3_RS09290) = 3,6-anhydro-L-galactose dehydrogenase.

[3]AHGI (VEJY3_RS09420) = 3,6-anhydro-L-galactose isomerase.

Supplementary Table 3: Primers used for RT-PCR of *np1*_1-33

Gene	Forward Primer (5' to 3')	Reverse Primer (5' to 3')
*np1*_1	AGCCAATTTGGAGAGAAACGA	CTTTCCTTTCTCCGCTATACCA
*np1*_2	CCTATCTATCGCACCCTCTCC	CACCATTTTTACCTTCGAGCA
*np1*_3	CAATACATGCTTTTGCCTCCT	CCTTGAGGTAAACAGTACGCTGA
*np1*_4	GGCATCTTGGTCTTGGTGATA	AAGCTCTTCCGTTTTCAATCC
*np1*_5	ATTGGGAAGAACAGGGGATAG	CGGTACTTCCGCTCATTACAG
*np1*_6	GGCAAGGAAAGGTGGTTTAAG	TCATCCCTGTCATTACTGTTGC
*np1*_7	TGGGGATATAATCCCAAAACG	ACCCTTGTACCCTTCACATCC
*np1*_8	CCGTTTCAGGACTATCAACCA	TCCCAGATACCCTTCTTACCA
*np1*_9	GCTTCGGAAGATCCTGTTGA	GGAATCATATTTTCCCCGTTG
*np1*_10	AGTTGAGAAAGGTGACGGTGA	TCCACTCTATCCCAACGTGTC
*np1*_11	GATTACGACAAAACGCGGTAA	CCACCTAAAGCACGCTGATAC
*np1*_12	GGGTGTGGAAAACAGACTTGA	TACAGCGTTGAATGCCAGATA
*np1*_13	TAATGGATTGTTCCGATGAGG	CTTGCACCAATGCTCCTAAAA
*np1*_14	AGAATGGAAGCCAACATTTCC	CGCTAATCTTCCCAATAATCC
*np1*_15	CGAGGAGTCGTCCCTAAATTC	CGGGAAATAAGGTTGGGTATT
*np1*_16	CTGTCCATAGGAACCGAAAGA	CAGCAGGTCTTATCCATTTGC
*np1*_17	GGAGACTAACCAGTCGGAGGA	CAAGACCTTCCTTTCCGTTTC
*np1*_18	TTCAGTGGGGATAGTGACTGG	CAGCCCGTTCTTTGTAGTCAG
*np1*_19	GGAAGAAAAATGGGGTGAGAA	GACGATGATATCCCTCGACTG
*np1*_20	CCTGCAAACTGGAAAGATGAG	GCCCATTGTCTTTTCGTCATA
*np1*_21	CTCATCAACGTTTTGGCTTTC	TTTCCGTTGAAATCACCAGTC
*np1*_22	ACGTAGGACCAAAAGTTGACG	GTTCCCTTGTTTTCTCCTTCG
*np1*_23	GTAATAACTGCCGATGGCAAA	TGCAGCATTACCGTTTCCTAC
*np1*_24	CGACAATGAAGAAACTGGTGAC	CCATCCTCACACAAGCCTTTA
*np1*_25	ATTACCCCAGCCATTTCTGAT	ATGGACGCATAATGGACTGAG
*np1*_26	GGCTGTTGTAACACAGGGAAA	TTCTTAAACAGACCCCACGTC
*np1*_27	GGGGTTCCCTGGTAATGTAAC	CTCCATAAATCCCGAAAGATTG
*np1*_28	TGCTTCATTCGCAATTTCTG	GTCCAAAGGGAACAGAAGGTC
*np1*_29	CAGTTGGAGTTGGGAGGAAAG	CTCGTGAAGATACAGGCGTTC
*np1*_30	AAAGGATATGGTGGCAAGAGG	TTACCAGCCCATTCATTAGCA
*np1*_31	CCGGTGTTCAGAGTACGGTTA	ACACGAGCTCCCATGAATGTA
*np1*_32	TCCCCGATTCTCCTCTAACAT	TCCGGTACGATAGGGTAATCC
*np1*_33	AAGCGGATTCTTTGGGAATAC	CCTTCCTCATAAGCCAAATCA

Supplementary Table 4: Primers used for RT-PCR of housekeeper genes

Gene	Forward Primer (5' to 3')	Reverse Primer (5' to 3')
50S Ribosomal Protein L17	GTCCGGGTGGTTACACTCGT	AGCTTCCTCAGCAGCAGGAG
50S Ribosomal Protein L9	AAAGAAGACATTGTAAACTTGGG	GATACGCCTTCCAGCTTAGC
Malonyl CoA-ACP transacylase	GTTTGCGTGGCAGCCAACTA	ATGGATTTCTGTTGCATTGA
Phosphoglycerate Kinase	TTATTGCCGGCGGTATGACT	GGGCAGAACTTGGTGTTGGC
RNA Polymerase σ Factor rpoD (σ-A)	TCCGTTGAACCAGGTAGGCT	AGCTGTTGTCCTCTCCTTCT
Signal Recognition Protein	GCTATCGAGCAGTTGCGTGT	CGGCAATCTCGTTCATCATC
UDP-3-O-[3-Hydroxymyristoyl] GlcNAc Deacetylase	CAAGCCGTACTTTCGTGTTT	AGGTTTGTGGTTGATATATCC
Bt/Bo_16S	AGGTTTGTGGTTGATATATCC	GGTAGTCCACACAGTAAACGATGAA
Bt16S1	GGTAGTCCACACAGTAAACGATGAA	CCCGTCAATTCCTTTGAGTTTC

Supplementary Table 5: Primer sequences used to recombinant *Bu* NP1 Ag-PUL proteins (*NdeI or NheI* and *XhoI)* restriction sites are underlined

Primer	Sequence 5' -> 3'
GH2A_Forward	TTT GGA GCT AGC GTA ACG GAT GGT ATC TCC TTC
GH2A_Reverse	CTT TGT CTC GAG TTA AAG TTC TAC ATT TAT TGT TGT C
GH2B_Forward	TTT GGA GCT AGC CAG CCG GGC TTC GAA TCA TTT TC
GH2B_Reverse	CTT TGT CTC GAG CTA TTT AGC TTT TCC AAA ACC TTT GG
GH2C_Forward	TTT GGA GCT AGC TAT GCG TTA AAT AAT AAT TCC G
GH2C_Reverse	CTT TGT CTC GAG TCA TTT AAC TTC AAC CTT TAA ATC
GH16A_Forward	TTT GGA GCT AGC CAA AAC CTT CCG GAG GTT CCG
GH16A_Reverse	CTT TGT CTC GAG TCA GTC TAC ATA CAT AGT ATT G
GH16B_Forward	TTT GGA GCT AGC GGA GAT AAT AAT GGA GAA GAG
GH16B_Reverse	CTT TGT CTC GAG TTA TTT ATC CTC TAC CGG TTT ATA AAT TC
GH16C_Forward	TTT GGA GCT AGC AAT GAA GTT ATT ATA GAA ACA GG
GH16C_Reverse	CTT TGT CTC GAG TTA TAA AGT TTT CAA AAT ATA TG
GH86_Forward	TTT GGA GCT AGC CAG ACT CAT GTG CAA TTG AAT TTG
GH86_Reverse	CTT TGT CTC GAG TCA TAT ACC TCT AAT ATT ATT TG
GH86_E322Q_Fwd	CCT CTT ATG ATC TCG CAA TAC AGT GCG
GH86_E322Q_Rev	C ATG CGT CTG CGC ACT ATG TTG CG
GH117A_Forward	TTT GGA GCT AGC CAG AAT CCA TTC CCT TAT G
GH117A_Reverse	CTT TGT CTC GAG TTA GAT AAT CTC CTT CTG CTT TTT AG
GH117B_Forward	TTT GGA GCT AGC ACA GTG TCT ACT GAT GAT AGT GC
GH117B_Reverse	CTT TGT CTC GAG TTA TTT TCG CTT TGT TGA TTC ACC
NP1_9_Forward	TTA AGC TAG CTG CCA AAA GGA CTA TGA GTT TAA C
NP1_9_Reverse	TTA ACT CGA GCT ATG GAC GTT TTT CTA TTT TAG TC
NP1_10_Forward	TTA AGC TAG CTG TGA AGA TGC CTT GCT GTC AC
NP1_10_Reverse	TTA ACT CGA GTT AAT TCT GTA ATG CTT TAT TAT TCT G

Supplementary Table 6: Characterized enzyme activities from Ag-PUL

Glycoside Hydrolase	Agarase
GH2A	-
GH2B	-
GH2C	+
GH16A	-
GH16B	+
GH16C	-
GH29	-
GH86	+
GH117A	-
GH117B	+

Supplementary Table 7: X-ray data collection and statistics of apo structures

	GH2C	GH16B	GH86	GH117B
Data Collection				
Beamline	CLS - 08ID-1	CLS - 08ID-1	CLS - 08ID-1	CLS - 08ID-1
Wavelength (Å)	1.0000	1.28215	0.97949	0.97949
Space Group	P1	$P4_32_12$	$I2_3$	$P2_12_12_1$
Cell Dimensions				
a, b, c (Å)	75.1, 121.4, 124.5	109.7, 109.7, 239.3	167.7, 167.7, 167.7	84.3, 100.3, 112.7
α, β, γ (°)	70.9, 87.5, 79.0	90.0, 90.0, 90.0	90.0, 90.0, 90.0	90.0, 90.0, 90.0
Resolution (Å)[1]	73.73-2.50 (2.64-2.50)	80.00-2.50 (2.64-2.50)	53.02-2.30 (2.42-2.30)	65.00-2.35 (2.48-2.35)
R_{merge}	0.121 (0.548)	0.13 (0.549)	0.109 (0.566)	0.147 (0.463)
R_{pim}	0.104 (0.475)	0.076 (0.327)	0.034 (0.177)	0.059 (0.186)
CC(1/2)	0.935 (0.298)	0.987 (0.721)	0.997 (0.916)	0.993 (0.886)
$\langle I/\sigma I \rangle$	5.7 (2.4)	7.6 (2.7)	14.8 (4.7)	11.6 (4.2)
Completeness (%)	97.8 (97.1)	99.7 (100.0)	100.0 (100.0)	99.6 (98.4)
Redundancy	2.2 (2.2)	3.6 (3.6)	11.2 (11.2)	7.1 (7.0)
No. of reflections	303,722	181,493	395,865	285,291
No. Unique	137,926	50,683	34,836	40,335
Refinement				
Resolution (Å)	2.50	2.50	2.30	2.35
R_{work}/R_{free}	0.21/.026	0.17/0.21	0.17/0.21	0.16/0.20
No. of atoms				
Protein	6508 (A), 6494 (B), 6451 (C), 6530 (D)	2428 (A), 2400 (B), 2383 (C)	5069	2978 (A), 2996 (B)
Ligand	10 (SO4), 28 (EDO)	3 (NA), 15 (IMD)	3 (CA), 6 (GOL), 24 (EDO)	2 (MG), 44 (EDO), 16 (TRS)
Water	755	394	261	590
B-factors				
Protein	29.9 (A), 30.3 (B), 35.2 (C), 28.3 (D)	31.0 (A), 31.2 (B), 33.0 (C)	43.5	18.3 (A), 18.4 (B)
Ligand	56.3 (SO4), 41.2 (EDO)	18.0 (NA), 49.2 (IMD)	38.1 (CA), 58.5 (GOL), 53.2 (EDO)	13.0 (MG), 41.6 (EDO), 22.2 (TRS)
Water	22.9	30.3	42.2	21.2
r.m.s.d				
Bond lengths (Å)	0.008	0.012	0.010	0.007
Bond angles (°)	1.195	1.604	1.340	1.296
Ramachandran (%)				
Preferred	95.5	96.9	95.7	98.1
Allowed	4.5	3.1	4.1	1.6
Disallowed	0.0	0.0	0.2	0.3
PDB code	5T9A	5T9X	5TA1	5TA7

[1]Values for highest resolution shells are shown in parenthesis.

Supplementary Table 8: X-ray data collection and statistics of agarase complexes

	GH2C GIF complex	GH86 E322Q N8 complex	GH117B N2
Data Collection			
Beamline	SSRL BL7-1	CLS - 08ID-1	CLS - 08ID-1
Wavelength (Å)	0.9753	0.9840	0.97949
Space Group	P1	P1	$P2_12_12_1$
Cell Dimensions			
a, b, c (Å)	75.2, 121.1, 124.5	60.7, 73.5, 83.2	83.5, 104.4, 198.5
α, β, γ (°)	70.7, 86.8, 78.8	85.9, 86.2, 71.9	90.0, 90.0, 90.0
Resolution (Å)[1]	73.79-2.40 (2.53-2.40)	48.39-1.40 (1.48-1.40)	76.97-2.40 (2.53-2.40)
R_{merge}	0.142 (0.638)	0.070 (0.324)	0.160 (0.540)
R_{pim}	0.085 (0.383)	0.029 (0.136)	0.059 (0.193)
CC(1/2)	0.990 (0.786)	0.998 (0.952)	0.989 (0.825)
<I/[illegible]I>	8.1 (2.0)	15.7 (5.4)	8.2 (3.6)
Completeness (%)	87.6 (82.5)	95.7 (93.4)	96.0 (90.0)
Redundancy	3.6 (3.4)	6.6 (6.6)	8.3 (8.6)
No. of reflections	504,239	1,705,176	543,181
No. Unique	139,311	256,936	65,697
Refinement			
Resolution (Å)	2.40	1.40	2.40
R_{work}/R_{free}	0.21/0.26	0.14/0.16	0.21/0.25
No. of atoms			
Protein	6533 (A), 6496 (B), 6554 (C), 6547 (D)	5413 (A), 5292 (B)	2970 (A), 2963 (B), 2935 (C), 2930 (D)
Ligand	40 (GIF), 56 (EDO)	40 (SO4), 120 (EDO), 4 (CA), 54 (N8), 54 (GOL)	48 (GAL), 44 (AAL), 4 (MG)
Water	851	1312	447
B-factors			
Protein	30.8 (A), 33.8 (B), 30.5 (C), 30.5 (D)	12.3 (A), 13.5 (B)	37.3 (A), 36.0 (B), 48.4 (C), 49.3 (D)
Ligand	29.0 (GIF), 39.7 (EDO)	34.4 (SO4), 28.8 (EDO), 10.4 (CA) 21.6 (N8), 29.8 (GOL)	41.2 (GAL), 51.7 (AAL), 26.0 (MG)
Water	22.2	25.8	32.0
r.m.s.d			
Bond lengths (Å)	0.009	0.010	0.008
Bond angles (°)	1.292	1.488	1.304
Ramachandran (%)			
Preferred	96.3	96.7	96.8
Allowed	3.6	3.1	3.1
Disallowed	0.1	0.2	0.1
PDB code	5T9G	5TA0	5TA9

[1]Values for highest resolution shells are shown in parenthesis.

Supplementary References

1. Loytynoja A & Goldman N (2005) An algorithm for progressive multiple alignment of sequences with insertions. *Proceedings of the National Academy of Sciences of the United States of America* 102(30):10557-10562.
2. Pluvinage B, Hehemann JH, & Boraston AB (2013) Substrate recognition and hydrolysis by a family 50 exo-beta-agarase, Aga50D, from the marine bacterium Saccharophagus degradans. *The Journal of biological chemistry* 288(39):28078-28088.
3. Rebuffet E*, et al.* (2011) Discovery and structural characterization of a novel glycosidase family of marine origin. *Environmental microbiology* 13(5):1253-1270.
4. Gasteiger E*, et al.* (2003) ExPASy: The proteomics server for in-depth protein knowledge and analysis. *Nucleic acids research* 31(13):3784-3788.
5. Jackson P (1993) Fluorophore-assisted carbohydrate electrophoresis: a new technology for the analysis of glycans. *Biochemical Society transactions* 21(1):121-125.
6. Powell HR (1999) The Rossmann Fourier autoindexing algorithm in MOSFLM. *Acta crystallographica. Section D, Biological crystallography* 55(Pt 10):1690-1695.
7. Collaborative Computational Project N (1994) The CCP4 suite: programs for protein crystallography. *Acta crystallographica. Section D, Biological crystallography* 50(Pt 5):760-763.
8. McCoy AJ*, et al.* (2007) Phaser crystallographic software. *Journal of applied crystallography* 40(Pt 4):658-674.
9. Cowtan K (2006) The Buccaneer software for automated model building. 1. Tracing protein chains. *Acta crystallographica. Section D, Biological crystallography* 62(Pt 9):1002-1011.
10. Emsley P & Cowtan K (2004) Coot: model-building tools for molecular graphics. *Acta crystallographica. Section D, Biological crystallography* 60(Pt 12 Pt 1):2126-2132.
11. Murshudov GN, Vagin AA, & Dodson EJ (1997) Refinement of macromolecular structures by the maximum-likelihood method. *Acta crystallographica. Section D, Biological crystallography* 53(Pt 3):240-255.
12. Brunger AT (1992) Free R value: a novel statistical quantity for assessing the accuracy of crystal structures. *Nature* 355(6359):472-475.
13. Davis IW*, et al.* (2007) MolProbity: all-atom contacts and structure validation for proteins and nucleic acids. *Nucleic acids research* 35(Web Server issue):W375-383.
14. Chen VB*, et al.* (2010) MolProbity: all-atom structure validation for macromolecular crystallography. *Acta crystallographica. Section D, Biological crystallography* 66(Pt 1):12-21.
15. Hehemann JH, Kelly AG, Pudlo NA, Martens EC, & Boraston AB (2012) Bacteria of the human gut microbiome catabolize red seaweed glycans with carbohydrate-active enzyme updates from extrinsic microbes. *Proceedings of the National Academy of Sciences of the United States of America* 109(48):19786-19791.
16. Hehemann JH, Smyth L, Yadav A, Vocadlo DJ, & Boraston AB (2012) Analysis of keystone enzyme in Agar hydrolysis provides insight into the degradation (of a polysaccharide from) red seaweeds. *The Journal of biological chemistry* 287(17):13985-13995.
17. Lombard V, Golaconda Ramulu H, Drula E, Coutinho PM, & Henrissat B (2014) The carbohydrate-active enzymes database (CAZy) in 2013. *Nucleic acids research* 42(Database issue):D490-495.
18. Yin Y*, et al.* (2012) dbCAN: a web resource for automated carbohydrate-active enzyme annotation. *Nucleic acids research* 40(Web Server issue):W445-451.
19. Edgar RC (2004) MUSCLE: multiple sequence alignment with high accuracy and high throughput. *Nucleic acids research* 32(5):1792-1797.

20. Stamatakis A (2014) RAxML version 8: a tool for phylogenetic analysis and post-analysis of large phylogenies. *Bioinformatics* 30(9):1312-1313.
21. Darriba D, Taboada GL, Doallo R, & Posada D (2011) ProtTest 3: fast selection of best-fit models of protein evolution. *Bioinformatics* 27(8):1164-1165.
22. Abbott DW & Boraston AB (2012) Quantitative approaches to the analysis of carbohydrate-binding module function. *Methods in enzymology* 510:211-231.
23. Hellemans J, Mortier G, De Paepe A, Speleman F, & Vandesompele J (2007) qBase relative quantification framework and software for management and automated analysis of real-time quantitative PCR data. *Genome biology* 8(2):R19.
24. Edgar RC (2004) MUSCLE: a multiple sequence alignment method with reduced time and space complexity. *BMC bioinformatics* 5:113.
25. Lee CH*, et al.* (2014) A novel agarolytic beta-galactosidase acts on agarooligosaccharides for complete hydrolysis of agarose into monomers. *Applied and environmental microbiology* 80(19):5965-5973.
26. Juncker AS*, et al.* (2003) Prediction of lipoprotein signal peptides in Gram-negative bacteria. *Protein science : a publication of the Protein Society* 12(8):1652-1662.
27. Seydel A, Gounon P, & Pugsley AP (1999) Testing the '+2 rule' for lipoprotein sorting in the Escherichia coli cell envelope with a new genetic selection. *Molecular microbiology* 34(4):810-821.
28. Yun EJ*, et al.* (2015) The novel catabolic pathway of 3,6-anhydro-L-galactose, the main component of red macroalgae, in a marine bacterium. *Environmental microbiology* 17(5):1677-1688.
